# Association of pulmonary, cardiovascular, and hematologic metrics with carbon nanotube and nanofiber exposure among U.S. workers: a cross-sectional study

**DOI:** 10.1186/s12989-018-0258-0

**Published:** 2018-05-16

**Authors:** Mary K. Schubauer-Berigan, Matthew M. Dahm, Aaron Erdely, John D. Beard, M. Eileen Birch, Douglas E. Evans, Joseph E. Fernback, Robert R. Mercer, Stephen J. Bertke, Tracy Eye, Marie A. de Perio

**Affiliations:** 1 National Institute for Occupational Safety and Health (NIOSH), Division of Surveillance, Hazard Evaluations, and Field Studies, 1090 Tusculum Ave MS-R15, Cincinnati, OH 45226 USA; 2 NIOSH, Health Effects Laboratory Division, Morgantown, WV USA; 3 Centers for Disease Control and Prevention, Epidemic Intelligence Service, Atlanta, GA USA; 4 NIOSH, Division of Applied Research and Technology, Cincinnati, OH USA; 5 Present address: Department of Public Health, College of Life Sciences, Brigham Young University, Provo, UT USA

**Keywords:** Epidemiology, Pulmonary function, Blood pressure, Heart rate, Occupational, Nanomaterials, Advanced manufacturing, Nanotoxicology

## Abstract

**Background:**

Commercial use of carbon nanotubes and nanofibers (CNT/F) in composites and electronics is increasing; however, little is known about health effects among workers. We conducted a cross-sectional study among 108 workers at 12 U.S. CNT/F facilities. We evaluated chest symptoms or respiratory allergies since starting work with CNT/F, lung function, resting blood pressure (BP), resting heart rate (RHR), and complete blood count (CBC) components.

**Methods:**

We conducted multi-day, full-shift sampling to measure background-corrected elemental carbon (EC) and CNT/F structure count concentrations, and collected induced sputum to measure CNT/F in the respiratory tract. We measured (nonspecific) fine and ultrafine particulate matter mass and count concentrations. Concurrently, we conducted physical examinations, BP measurement, and spirometry, and collected whole blood. We evaluated associations between exposures and health measures, adjusting for confounders related to lifestyle and other occupational exposures.

**Results:**

CNT/F air concentrations were generally low, while 18% of participants had evidence of CNT/F in sputum. Respiratory allergy development was positively associated with inhalable EC (*p*=0.040) and number of years worked with CNT/F (*p*=0.008). No exposures were associated with spirometry-based metrics or pulmonary symptoms, nor were CNT/F-specific metrics related to BP or most CBC components. Systolic BP was positively associated with fine particulate matter (*p*-values: 0.015-0.054). RHR was positively associated with EC, at both the respirable (*p*=0.0074) and inhalable (*p*=0.0026) size fractions. Hematocrit was positively associated with the log of CNT/F structure counts (*p*=0.043).

**Conclusions:**

Most health measures were not associated with CNT/F. The positive associations between CNT/F exposure and respiratory allergies, RHR, and hematocrit counts may not be causal and require examination in other studies.

**Electronic supplementary material:**

The online version of this article (10.1186/s12989-018-0258-0) contains supplementary material, which is available to authorized users.

## Background

Carbon nanotubes and nanofibers (CNT/F) are small, high-aspect-ratio (>5 μm long, <100 nm diameter) engineered nanomaterials of increasing commercial importance; e.g., they are used in conductive film and ink development in the electronics industry, and as strong, lightweight components of composites used in aircraft. Toxicological evidence suggests possible health effects from exposure to CNT/F, which were among the first engineered nanomaterials to reach commercialization [1]. Pulmonary inflammation and fibrotic changes or malignant transformation have been observed in animal models [2–4], as well as immunological, neurological, and cardiovascular effects, due to particle translocation or response to an inflammatory cascade [5–9].

Pulmonary function has been studied among workers exposed to engineered nanomaterials [10–12], and lung fibrosis was identified as the primary target outcome in a toxicology-based risk assessment for CNT/F [13]. However, the cardiovascular system may be more sensitive to adverse effects of engineered nanomaterials [14, 15], given findings from ambient ultrafine and fine particulate (U/FP) studies. Resting heart rate (RHR), a marker of changes in the autonomic nervous system, has been related to particulate air pollution [16] and found to be predictive of overall mortality risk [17]. Hypertension has been associated with ambient U/FP exposure in humans [18, 19]. Systemic inflammation can also be assessed by hematologic measures: increased neutrophil counts following exposure have been observed in welders [20]. Increased total leukocyte and neutrophil counts were found to be associated with ambient U/FP exposure in humans [18, 21] and with multiwalled CNT (MWCNT) in an animal model [9, 22].

The aim of this analysis was to evaluate whether there are associations between health-relevant metrics and exposure to CNT/F in a cross-sectional population of 108 U.S. workers. We did not employ an explicit “exposed” and “control” group design, but considered exposure-outcome associations across the full range of exposure in the study group. This approach both provides more statistical power and is more useful for risk assessment. We examined (1) self-reported respiratory illness or symptoms (after initiation of work with CNT/F); (2) spirometric lung function metrics; (3) cardiovascular health metrics; and (4) hematologic metrics. Lung function metrics were forced vital capacity (FVC) as one measure of potential restrictive lung disease, the ratio of forced expiratory volume in the first second (FEV1) to FVC and peak expiratory flow (PEF) as two metrics of potential obstructive lung disease, and forced expiratory flow at 25-75% of the pulmonary volume (FEF25-75%) as a potential indicator of small airways disease. Cardiovascular metrics were resting systolic and diastolic blood pressure (BP) and RHR. Hematologic measures were leukocyte counts and absolute concentrations of three leukocyte subcomponents (neutrophils, lymphocytes, and monocytes), platelets, hemoglobin, and hematocrit. Analyses of other markers of early effect (e.g., inflammatory cytokines, oxidative stress biomarkers, and endothelial activation products) in blood and sputum collected from this population are described elsewhere [23].

## Methods

### Selection of companies and participants

U.S. facilities handling CNT/F were eligible for inclusion in this study. Typical properties of CNT/Fs are described elsewhere [24]. We identified companies based on their participation in a survey to enumerate the engineered carbonaceous nanomaterial industry [1], supplemented with additional companies. Among 59 companies, 19 were considered for recruitment for the cross-sectional study, based on willingness to be interviewed about details related to their operations or participation in previous NIOSH exposure investigations [24, 25]. Four of the 19 companies were ineligible because they had stopped working with CNT/F (n=2) or were operating at purely research scale (n=2). Three eligible companies refused to participate. Thus, 80% of invited companies agreed to participate. For 11 of the 12 participating facilities, all employees working in a CNT/F unit were invited to participate. For one facility, due to its large size and study feasibility limitations, a subset of employees representing the widest variety of tasks was invited. Overall, 75% of eligible workers participated in the study (see Additional file 1: Table S1).

### Exposure assessment for CNT/F and U/FP

We visited participating companies from 12/2012-9/2014 and conducted at least two days of full-shift, personal breathing zone exposure monitoring for each study participant (except one participant who was unavailable during the period of exposure monitoring and was assigned the exposure level of a co-worker performing similar tasks), as described elsewhere [24, 26]. In summary, each participant wore three air sampling pumps connected to filters located in the participant’s breathing zone to measure the mass concentration of elemental carbon (EC) [13], for both the respirable and inhalable size fractions. A third set of air samples was collected for examination using transmission electron microscopy (TEM), which allowed the enumeration and size-binning of each particle with associated CNT/F, referred to as a CNT/F “structure” [27]. Air concentrations were calculated for total CNT/F structures (total structures/cm^3^), and for size-specific structures [single fibers/cm^3^, structures <1 μm (in diameter)/cm^3^, structures <2 μm/cm^3^, structures <5 μm/cm^3^, and structures <10 μm/cm^3^]. We evaluated multiple size bins for the structure counts because the most relevant size class of CNT/F for each health outcome is uncertain.

We used three direct-reading instruments to collect total ambient U/FP and ultrafine particulate (UP) count and U/FP mass, using general area samples contemporaneously collected with the CNT/F-specific measurements [26, 28, 29]. These metrics are: total U/FP counts (per cm^3^), defined as 10-1000 nm in diameter [collected with a condensation particle counter (CPC 3007; TSI, Inc., Shoreview, MN)], total UP particulate counts (per cm^3^), defined as 23-96 nm (collected with an electrical low-pressure impactor Dekati, Ltd, Tampere, Finland), and particulate mass (μg/m^3^) less than 2.5 μm (collected with a DustTrak® photometer; DRX Model 8533; TSI, Inc., Shoreview, MN). Estimates of each participant’s exposure were made, for each day of sampling, using professional judgment of the locations of the participant throughout their sampling day [26].

### Questionnaire administration

Mid-shift during a mid-week workday (concurrently with exposure assessment), each participant was administered a standardized questionnaire by a trained interviewer. Questions were included on demographics, medical history, current and past exposure to CNT/F and other physical and chemical agents, and smoking and alcohol consumption (see Additional file 2). Questions pertaining to respiratory symptoms and illnesses were obtained from the American Thoracic Society (ATS) 1978 Adult Questionnaire [30]. Questions related to fitness for undergoing spirometry were drawn from the National Health and Nutrition Examination Survey (NHANES) Respiratory Health Spirometry Procedures Manual [31].

### Medical examination, blood pressure and heart rate measurement, and spirometry

Immediately after questionnaire administration, participants were examined in a mobile examination unit by a study physician, who reviewed medical histories to determine eligibility for spirometry, sputum induction, and phlebotomy. Height without shoes (using a stadiometer) and waist circumference (using a tape measure) were measured to the nearest 0.5 cm, and weight was measured using a digital scale to the nearest 0.1 kg. Systolic and diastolic BP and RHR were ascertained after resting for five minutes, using the method described in NHANES [32, 33]. Readings were obtained with an OMRON™ HEM-907XL digital sphygmomanometer, which was calibration-checked weekly against an analog instrument. Three readings were collected for each metric, and the second and third readings were averaged for analysis. Two participants who completed the exposure assessment and questionnaire declined to participate in the medical examination and subsequent procedures.

We used a volume-based spirometer and standard methodology recommended by the ATS [34, 35] to collect spirometry metrics for all participants (*n*=103) not refusing or excluded based on medical exclusion criteria. Spirometry tests were conducted by a certified technician, who followed quality guidelines noted by ATS, and were interpreted clinically using ATS recommendations [35]. We used the percent predicted (PP) values for FVC, FEV1/FVC% (using the largest valid FEV1 and FVC), FEF25-75%, and PEF. Further details on the collection and interpretation of the spirometry data are provided in Additional file 3.

### Collection of whole blood and complete blood count measurement

Whole blood was obtained through venipuncture for the CBC analysis, as well as for serum and plasma biomarker analyses described elsewhere [23]. A 3-mL ethylene-diaminetetraacetic acid tube of whole blood was collected, inverted 10 times, and stored at room temperature until the end of the day, when each batch was sent to a clinical laboratory (Quest Diagnostics or LabCorp) for CBC analyses using an automated cell counter. A shipping error resulted in the loss of samples for four participants. CBC analyses were available for 98 participants.

### Sputum induction and processing

Sputum induction methodology is detailed in Additional file 3. In summary, seven participants were excluded based on contraindications. Ninety other participants agreed to provide sputum, which was induced by breathing aerosolized isotonic saline generated with a compressed-air nebulizer. Isotonic saline was used to reduce the likelihood of bronchial spasm induction and because previous research suggested that sputum of acceptable quality could be obtained [36]. However, we found the percentage of squamous epithelial cells, determined by manual counting, to be very high (>80% for all but one participant), likely due to the use of isotonic saline and the processing of the entire sputum specimen (rather than selecting sputum plugs from each specimen). Sputum cellular fractions were preserved at 4°C.

After arrival in the NIOSH laboratory, we prepared a cytospin of the cell pellet on ultrasonically cleaned, laser cut slides (Schott North America, Inc, Elmsford, NY). To enhance the contrast of nanomaterials, cytospin slides were stained with Sirius Red. Sections were briefly counterstained in freshly filtered Mayer’s hematoxylin for 2 minutes, dehydrated, and coverslipped. Approximately 3,000 cells per slide (<1% of each specimen) were examined for evidence of CNT/F structures using dark-field microscopy [37].

### Statistical methods

From the questionnaire, we estimated the length of time each participant worked with CNT/F (not always concordant with length of employment in the industry) as an integrative metric of past CNT/F exposure. We also estimated self-reported exposure to a variety of physical and chemical agents in the workplace (Additional file 1: Table S2). For most outcomes, the following were considered as potential confounders: age, sex, race/ethnicity, cigarette pack-years, self-reported current or past occupational exposures to solvents, polymers, strong acids, “other” (non-CNT/F) forms of nanomaterials, and a general category of particulates, termed “other dusts”. Other potential confounders for some outcomes included childhood pneumonia, current self-reported respiratory diseases, alcohol consumption, use of certain medications, and a modified cardiovascular health metric (CHM) score (after [38], except using six metrics: body mass index, waist circumference, hypertension diagnosis, diabetes diagnosis, cigarette smoking, and use of antihypercholesterolemic medication; see Additional file 1: Tables S3 and S4).

Five CNT/F exposure variables were evaluated in exposure-response analyses: respirable and inhalable EC mass concentrations in air, TEM structure concentrations in air, presence/absence of CNT structures in induced sputum, and duration of exposure to any form of CNT/F. All EC samples were background-corrected to account for other (naturally occurring and anthropogenic) sources of EC [24]. Arithmetic means of all sampled days were used for these metrics.

We used a prevalent case-control analysis for binary outcomes (i.e., self-reported chest symptoms and respiratory allergies) that permitted evaluation of whether the illness started before or after the start of work with CNT/F (e.g., [39]). We used logistic regression to model the odds of exposure among those who exhibited the outcome (cases) after the start of exposure to CNT/F compared to those who did not (controls), among those who were outcome-free before exposure began. Due to the small number of cases, we did not adjust for confounding for these outcomes, but we evaluated associations of potential confounders with the outcomes. For continuous metrics (i.e., systolic and diastolic BP, RHR, lung function metrics, CBC measures), we used multiple linear regression [40] to model the association between the exposure metrics and outcomes, adjusted for important confounders. Log-transforms (for FEF25-75%, leukocyte and its differential counts, and platelet counts) or square transforms (for hemoglobin and hematocrit concentrations) were used to improve normality of the model residuals. We evaluated log-transformation of the highly skewed CNT/F exposure metrics. Potential covariates were treated as continuous or categorical variables. Because some covariates were highly correlated, their inclusion could lead to poor parameter estimation [40], and the small sample size limited the number of covariates that could be included. Therefore, covariates were screened for each outcome metric in a model with no CNT/F metrics: the best-fitting model from among all possible combinations of up to 10 covariates was identified using Schwarz’s Bayesian Criterion (SBC), where a smaller SBC value indicates better model fit [41]. Use of SBC to select model predictors for outcomes in small studies has been shown to be superior to other methods at identifying the “true” underlying model, given correlated covariates [41]. Covariates so selected were retained if they changed the parameter estimate for the best-fitting CNT/F exposure metric by >10%. Model fit for alternative exposure metrics (e.g., log-transformed metrics; different TEM size bin cut points) was determined using SBC values. Parameter estimates and two-sided p-values were reported. CNT/F effect modification for each outcome was evaluated for sex, race/ethnicity (white non-Hispanic compared to all whites), age (<40 compared to ≥40), education level (college degree or higher compared to no college degree), smoking status (ever compared to never), reported respiratory disease or allergy, and CHM score (5-6 compared to 0-4). Fit was evaluated in the multiple regression models by evaluating residual patterns for heteroscedasticity or other fit problems [40]. All analyses were conducted using SAS ver. 9.4 (Cary, NC).

## Results

### Descriptive information

Table 1 shows the demographic characteristics of the study participants. The majority were male and of white race and non-Hispanic ethnicity. Age followed a bimodal distribution, with modes in the mid-30s and mid-50s. Most (65.8%) participants had at least a college degree, with 38% having a post-graduate education. Participants mostly reported never smoking cigarettes (63%) and currently drinking alcohol (65.7%).

**Table 1:** Demographic and lifestyle characteristics of 108 cross-sectional study participants

Characteristic	Group	N (%)
Sex	Male	85 (78.7%)
	Female	23 (21.3%)
Ethnicity and Race	Non-Hispanic White alone	87 (80.6%)
	Non-Hispanic Asian alone	10 (9.3%)
	African-American, American Indian/Alaska Native, Multiple races, and Hispanic combined	11 (10.2%)
Age (years)	<25	6 (5.6%)
	25-<35	33 (30.6%)
	35-<45	16 (14.8%)
	45-<55	28 (25.9%)
	55-<65	20 (18.5%)
	65-<75	5 (4.6%)
Highest education level	High school, Trade or vocational	13 (12.1%)
	Some college	24 (22.2%)
	College graduate	30 (27.8%)
	Postgraduate	41 (38.0%)
Cigarette smoking status	Never	68 (63.0%)
	Former	24 (22.2%)
	Current	16 (14.8%)
Alcohol consumption status	Never	7 (6.5%)
	Former	30 (27.8%)
	Current	71 (65.7%)

**Fig. 1:**
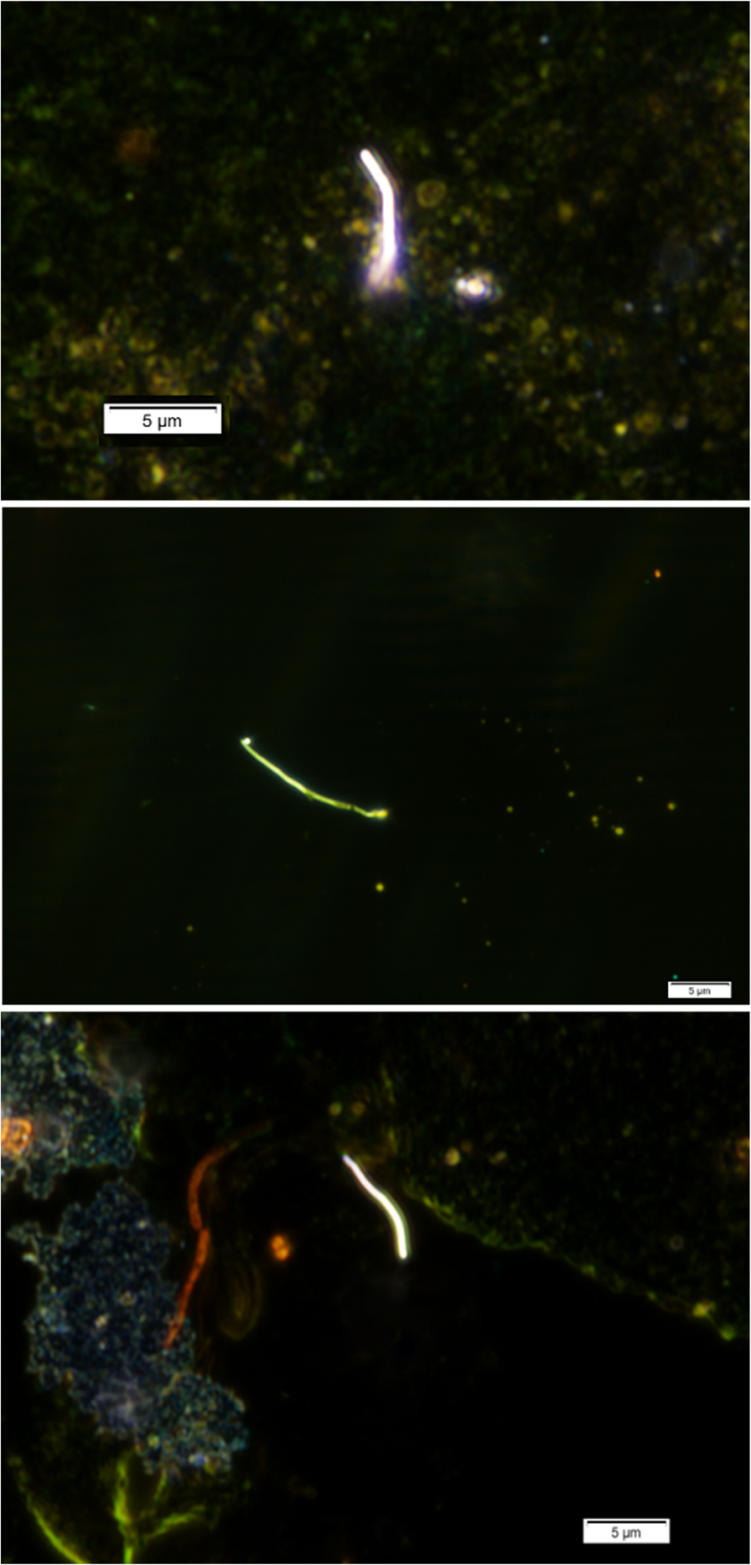
Dark-field microscopy images of carbon nanotube in biospecimen of sputum/saliva

Sputum was obtained from 90 participants. CNT/F was detected, via dark-field microscopy, in the sputum of 16 (17.7%) of these participants (typical images are provided in Fig. 1). Multi-day mean CNT/F exposure concentrations are shown in Table 2. Median concentrations for inhalable and respirable EC were low, at 0.24 and 0.096 μg/m^3^, respectively, while mean concentrations were substantially higher (6.22 and 1.00 μg/m^3^) due to a few outlying observations. Only 7 participants had background-corrected respirable EC levels above the NIOSH recommended exposure limit (REL) of 1 μg/m^3^. The mean and median TEM structure count concentrations (all sizes) were 0.128 and 0.0073 structures/cm^3^, respectively. Most structures (60%) were in the 2-10 μm size range, and just 20% of participants had any single fibers detected. Nearly all workers who directly worked with CNT/F handled them in unpurified form; for >80% of workers in our study, CNT/F had been manufactured with a Fe-based catalyst. The mean and median duration of time working with CNT/F were 4.07 and 3.66 years, respectively (Table 2). Sixteen participants (15%) indicated they had never worked with CNT/F.

**Table 2:** Descriptive statistics for carbon nanotube or nanofiber (CNT/F) exposure variables among 108 study participants

Exposure Variable^a^	Mean	Median	Standard deviation	25^th^, 75^th^ %-ile
*Multi-day mean EC (background-corrected) concentration (μg/m* ^*3*^ *)*				
Respirable size fraction	1.00	0.096	4.94	0.018, 0.33
Inhalable size fraction	6.22	0.24	41.2	0.027, 1.32
*Multi-day mean TEM structure count concentration (structures/cm* ^*3*^ *)*				
All CNT/F-containing structures	0.128	7.29E-3	0.469	9.02E-4, 8.30E-2
Structures < 10 μm diameter	0.121	6.03E-3	0.440	3.80E-4, 8.34E-2
Structures <5 μm diameter	0.0865	3.04E-3	0.316	9.60E-5, 6.52E-2
Structures <2 μm diameter	0.0443	3.08E-4	0.201	0, 3.33E-3
Structures <1 μm diameter	0.0398	0	0.194	0, 7.12E-4
Single fiber structures	0.0324	0	0.193	0, 0
Duration worked with CNT/F (years)	4.07	3.66	4.01	1.05, 5.61

^a^Abbreviations: *CNT/F* carbon nanotubes or nanofibers, *EC* elemental carbon, *TEM* transmission electron microscopy

### Pulmonary outcomes and metrics

A minority of study participants reported respiratory illnesses (allergy: 41%, asthma: 8%, COPD: 6%) or symptoms (42%) before their start of CNT/F work (Additional file 1: Table S5). No participants reported asthma or COPD after the start of CNT/F work, while 21% reported the initiation of one or more chest symptoms and 14% reported the development of respiratory allergies after beginning work with CNT/F (Table 3). 89% of those reporting respiratory allergies after the start of CNT/F work stated that their allergies had been confirmed by a physician. No CNT/F exposure metrics were significantly associated with development of chest symptoms after starting CNT/F work. No demographic, lifestyle, or occupational covariates were significantly associated with development of respiratory allergy (Additional file 1: Table S6), but both the inhalable EC concentration (OR=1.1 at mean exposure of 4 μg/m^3^; p=0.04) and duration of work with CNT/F (OR=1.2 at 1 year duration; p=0.008) were significantly positively associated with self-reported development of respiratory allergies (Table 3).

**Table 3:** Associations of occupational exposure metrics with chest symptom and respiratory allergy development among 108 workers

	Chest symptom	Respiratory allergy
N outcome-free at start of work	63	64
N (%) reporting outcome after start of CNT/F work	13 (21%)	9 (14%)
*Unadjusted OR (p-value* ^*a*^ *) from logistic regression*		
Duration of CNT/F work (OR at 1 year)	1.07 (0.36)	1.20 (0.0081)
Presence of CNT in sputum (OR yes:no)	NA^b^	0.91 (0.94)
EC – inhalable (OR at 1 μg/m^3^)	1.02 (0.34)	1.02 (0.040)
EC – respirable (OR at 1 μg/m^3^)	1.11 (0.19)	1.08 (0.096)
TEM structure count (OR at 0.1 structure/cm^3^)	1.03 (0.47)	1.07 (0.33)
Fine particulate counts (OR at 2000 per cm^3^)^c^	1.07 (0.31)	0.85 (0.095)
Nanoscale particulate counts (OR at 2000 per cm^3^)^d^	1.05 (0.63)	0.98 (0.85)
Fine particulate matter mass (OR at 10 μg/m^3^)^e^	0.84 (0.67)	0.61 (0.31)

Abbreviations: *CNT/F* carbon nanotubes or nanofibers, *EC* elemental carbon, *NA* not available, *OR* odds ratio, *TEM* transmission electron microscopy

^a^maximum-likelihood based

^b^no sputum CNT was detected among those reporting chest symptoms since start of CNT/F work

^c^measured with condensation particle counter (10-1000 nm diameter)

^d^measured with electrical low-pressure impactor (23-96 nm diameter)

^e^measured with photometer (<2.5 μm diameter)

Spirometry metrics indicated an overall healthy respiratory profile among participants (Table 4): 92 participants (89%) exhibited normal expiratory flows and a normal FVC. Five participants (4.9%) had restrictive patterns, and four participants (3.9%) showed obstructive patterns. No participants exhibited mixed obstructive and restrictive patterns. Results were not clinically interpretable for two participants (1.9%). Few covariates (namely, race/ethnicity, cigarette smoking, and current or past exposure to solvents, dust, or strong acids) were significantly related to any of the PP values for FVC, FEV1/FVC, FEF25-75%, or PEF (Additional file 1: Table S7). After adjusting for covariates, no lung function metric was significantly negatively associated with any of the CNT metrics (Table 5). However, duration of employment with CNT/F was positively associated with FEV1/FVC, FEF25-75%, and PEF. Use of a log-transform for the EC and TEM exposure metrics showed similar results. No significant effect modification was observed by sex, race, age, education, prior lung disease, or CHM score (Table 5).

**Table 4:** Descriptive statistics for outcome variables among 108 study participants

Outcome Variable	N available	Mean	Median	Standard deviation	25^th^-75^th^ %-ile	Min.-Max.	N (%) outside normal range^a^
*Spirometry measures*							
FVC PP	103	98.6%	99.6%	11.5%	90.7-105%	66.8-133%	5 (5.0%)
FEV1/FVC ratio PP	103	99.6%	100%	7.56%	93.9-105%	78.6-116%	8 (7.8%)^b^
FEF25-75 PP	103	100%	96.7%	31.5%	75.0-125%	35.6-185%	8 (7.8%)
PEF PP	103	107%	107%	14.6%	96.9-117%	69.8-142%	1 (1.0%)
*Cardiovascular measures*							
Systolic BP (mmHg)	106	122	122	14.2	112-130	92-164	13 (12.3%)
Diastolic BP (mmHg)	106	75.1	74.5	10.1	69-82	42.5-106	6 (5.7%)
Heart rate (BPM)	106	69.9	69.8	11.2	61.5-77	45.5-108	1 (0.9%)
*Blood component measures*							
Leukocyte count (x10^3^/μL)	98	7.01	6.80	1.70	5.9-8.0	3.5-12.2	2 (2.0%)
Abs. neutrophils	98	3.99	3.80	1.24	3.2-4.7	1.6-7.3	1 (1.0%)
Abs. lymphocytes	98	2.33	2.30	0.69	1.8-2.6	1.1-4.8	8 (8.2%)^c^
Abs. monocytes	98	0.50	0.50	0.15	0.4-0.6	0.20-0.83	2 (2.0%)
Platelet (x10^3^/μL)	98	249.8	238.5	53.2	212-279	157-460	1 (1.0%)
Hemoglobin (g/dL)	98	15.1	15.3	1.18	14.3-16	12.1-17.5	2 (2.0%)
Hematocrit	98	44.8%	45.1%	2.98%	42.5-47.3%	37.3-49.9%	0

Abbreviations: *BP* blood pressure, *BPM* beats per minute, *FEV1* forced expiratory volume in one second, *FVC* forced vital capacity, *PP* percent predicted, *FEF25-75* forced expiratory flow at 25-75-% of the pulmonary volume, *PEF* peak expiratory flow

^a^Below the lower limit of normal for pulmonary function measures (two subjects’ tests were not interpretable with respect to this criterion); above 139 and 89 mm Hg for systolic and diastolic (respectively) BP; above 100 beats per minute for heart rate; above or below the reference range for blood component measures

^b^Includes three participants determined clinically to have normal spirometry pattern and one participant with clinically uninterpretable spirometry

^c^All were above the reference range

**Table 5:** Results of multiple linear regression modeling of pulmonary function metrics

	CNT/F metric β estimate (*p-*value)			
	FVC PP^a^	FEV1/FVC PP^b^	ln(FEF25-75 PP)^c^	PEF PP^d^
*Exposure variable - untransformed*				
EC–inhalable (μg/m^3^)	-1.78E-4 (0.478)	2.53E-4 (0.155)^e^	2.65E-4 (0.754)	8.17E-5 (0.810)
EC-respirable (μg/m^3^)	-6.12E-4 (0.772)	1.87E-3 (0.208)	2.12E-3 (0.771)	1.76E-4 (0.950)
TEM-total (structures/cm^3^)	1.02E-2 (0.651)	-4.80E-4 (0.976)	3.97E-2 (0.555)	-2.54E-2 (0.398)
CNT/F found in sputum	-1.61E-2 (0.558)	-5.15E-3 (0.804)	-3.14E-2 (0.707)	1.57E-2 (0.700)
CNT/F duration employed (years)	-2.30E-4 (0.930)	2.83E-3 (0.125)	1.98E-2 (0.0148)	5.32E-3 (0.130)
*Exposure variable – log-transformed*				
ln(EC-inhalable)	1.63E-6 (0.999)	1.27E-3 (0.598)	1.08E-2 (0.288)	-4.41E-3 (0.334)
ln(EC-respirable)	6.79E-3 (0.172)^e^	6.28E-4 (0.857)	1.77E-2 (0.224)	-9.24E-3 (0.159)^e^
ln(TEM-total)	5.34E-3 (0.203)	2.55E-3 (0.376)	2.14E-2 (0.0746)^e^	3.23E-3 (0.554)
*Effect modification* ^*f*^				
Male	6.86E-3	2.51E-4	2.27E-2	-9.40E-3
Female	6.55E-3	6.12E-4	1.73E-2	-8.72E-3
*p* for interaction	0.968	0.869	0.689	0.948
White race, non-Hispanic	8.02E-3	2.50E-4	2.04E-2	-7.76E-3
All other races and Hispanic	-8.48E-4	-1.53E-3	2.83E-2	-1.54E-2
*p* for interaction	0.533	0.907	0.592	0.496
Age <40	3.81E-3	-1.32E-4	1.35E-2	-1.16E-2
Age ≥40	8.66E-3	2.74E-4	2.56E-2	-7.41E-3
*p* for interaction	0.468	0.587	0.304	0.635
Education < college degree	1.11E-2	-1.65E-3	3.09E-2	-1.83E-3
Education ≥ college degree	5.57E-3	2.80E-4	1.58E-2	-1.11E-2
*p* for interaction	0.457	0.149	0.212	0.349
No lung disease^g^	6.38E-3	1.69E-4	1.84E-2	-1.82E-2
Has lung disease	6.99E-3	2.56E-4	2.23E-2	-3.90E-3
*p* for interaction	0.927	0.928	0.726	0.0985
CHM score <5	7.91E-4	2.54E-4	3.00E-2	-9.74E-3
CHM score ≥5	1.41E-2	3.03E-6	1.80E-2	-8.87E-3
*p* for interaction	0.186	0.908	0.287	0.922

Abbreviations— *CHM* cardiovascular health metric, *CNT/F* carbon nanotubes or nanofibers, *EC* elemental carbon, *FEV1* forced expiratory volume in one second, *FVC* forced vital capacity, *FEF25-75* forced expiratory fraction between 25 and 75% of maximal, *PEF* peak expiratory flow, *PP* percent predicted, *TEM* transmission electron microscopy

^a^FVC percent predicted adjusted for race/ethnicity, high CHM score, and self-reported current exposure to strong acids

^b^FEV1/FVC percent predicted unadjusted

^c^FEF25-75% adjusted for cigarette pack-years, self-reported current solvent exposure, and duration of exposure to CNT/F

^d^PEF adjusted for self-reported past exposure to dust

^e^CNT/F exposure metric associated with the lowest Schwarz’s Bayesian Criterion value (best fit)

^f^Parameter estimates and p-values are shown for the CNT/F exposure metric identified as having the best fit

^g^Self-reported respiratory allergy, asthma, or chronic obstructive pulmonary disease

### Cardiovascular metrics

A majority (58%) of subjects had BP in a range associated with pre-hypertension or hypertension (i.e., systolic BP ≥120 or diastolic BP ≥80), while 13% had BP in a range associated with hypertension (i.e., systolic BP ≥140 or diastolic BP ≥90) (Table 4). Several covariates were significantly positively associated with systolic BP, diastolic BP, or RHR (Additional file 1: Table S8). U/FP counts were significantly positively associated only with systolic BP. Both before and after adjusting for confounding, none of the CNT/F metrics was significantly associated with systolic or diastolic BP, and point estimates tended to be negative for most exposure metrics (Additional file 1: Table S6 and S8). After adjusting for confounding, RHR was significantly positively associated with inhalable and respirable EC and significantly negatively associated with duration of time worked with CNT/F. (Table 6). Use of a log-transform for the EC and TEM exposure metrics showed similar results except for respirable EC, which was no longer significantly associated with RHR. While no significant effect modification was observed for BP by age, sex, race, education, prior lung disease, or CHM score, we found a significant interaction between inhalable EC and sex (p=0.063) for RHR: its positive association with EC was restricted to men (Table 6).

**Table 6:** Results of multiple linear regression modeling of cardiovascular metrics

	CNT/F metric β estimate (*p*-value)		
	Systolic BP^a^	Diastolic BP^b^	Heart rate^c^
*Exposure variable – untransformed*			
EC–inhalable (μg/m^3^)	-2.65E-2 (0.386)	-2.85E-2 (0.213)^d^	8.63E-2 (0.0026)^d^
EC-respirable (μg/m^3^)	-0.255 (0.317)^d^	-0.221 (0.251)	0.667 (0.0074)
TEM-total (structures/cm^3^)	-0.321 (0.905)	-0.992 (0.624)	0.929 (0.688)
CNT/F found in sputum	1.03 (0.779)	1.02 (0.712)	1.20 (0.695)
CNT/F duration employed (years)	0.181 (0.575)	-9.46E-3 (0.968)	-0.777 (0.0029)
*Exposure variable – log-transformed*			
ln(EC-inhalable)	0.187 (0.653)	-5.72E-2 (0.856)	0.958 (0.0055)
ln(EC-respirable)	-0.588 (0.331)	-0.367 (0.416)	0.190 (0.705)
ln(TEM-total)	0.349 (0.469)	-2.59E-4 (0.999)	0.216 (0.597)
*Effect modification* ^*e*^			
Male	-0.238	-0.0270	0.0870
Female	-3.52	-0.241	-0.454
*p* for interaction	0.350	0.459	0.063
White race, non-Hispanic	-0.259	-0.0284	0.0860
All other races and Hispanic	-11.6	0.417	-1.81
*p* for interaction	0.398	0.819	0.352
Age <40	-0.195	-5.88E-2	1.55E-2
Age ≥40	-0.279	-2.69E-2	8.79E-2
*p* for interaction	0.881	0.740	0.483
Education < college degree	-0.206	-6.38E-2	9.86E-2
Education ≥ college degree	-0.257	-2.81E-2	8.60E-2
*p* for interaction	0.966	0.837	0.945
No lung disease^f^	-0.254	-7.14E-4	9.03E-2
Has lung disease	-0.255	-2.94E-2	8.63E-2
*p* for interaction	1.00	0.814	0.976
CHM score <5	-0.254	-0.0263	0.0867
CHM score ≥5	-5.49	-0.315	-0.383
*p* for interaction	0.133	0.309	0.112

Abbreviations—*BP* blood pressure, *CHM* cardiovascular health metric, *CNT/F* carbon nanotubes or nanofibers, *EC* elemental carbon, *TEM* transmission electron microscopy

^a^Systolic BP adjusted for age, sex, and total 10-1000 nm particulate counts per cm^3^ (measured with condensation particle counter)

^b^Diastolic BP adjusted for sex, cigarette pack-years, and CHM score

^c^Heart rate adjusted for employment duration and CHM score

^d^CNT/F exposure metric associated with the lowest Schwarz’s Bayesian Criterion value (best fit)

^e^Parameter estimates and *p*-values are shown for the CNT/F exposure metric identified as having the best fit

^f^Self-reported respiratory allergy, asthma, or COPD

### Hematologic metrics

Most participants had blood component counts within the normal range (Table 4); however, absolute lymphocyte count was elevated for 8.2% of participants. U/FP count concentration was significantly positively associated with total leukocyte, neutrophil and lymphocyte counts (Additional file 1: Table S9). Several covariates (including sex, race/ethnicity, alcohol consumption, current respiratory infection, CHM score, and past exposure to solvents or “other” nanomaterials) were significantly associated with one or more blood components (Additional file 1: Tables S9 and S10). After adjusting for confounding, none of the CNT/F metrics was significantly associated with leukocyte counts or platelets. Log-transformed TEM structure concentration was positively associated with hematocrit (p=0.04) (Table 7). Significant interactions were seen with TEM structure concentrations and race/ethnicity (Hispanic and non-white participants had a stronger negative association between TEM structure concentrations and both leukocytes and neutrophils than other participants) and with the log of TEM structure counts and education (participants with college degrees had a stronger positive relationship between the log of TEM structure counts and both hemoglobin and hematocrit than other participants; Table 7). There were also significant interactions between the log of TEM structure counts and sex (females had stronger negative associations between the log of TEM structure counts and platelets than males), age (participants less than age 40 years had stronger negative associations between the log of TEM structure counts and platelets than participants age 40 years or older), and CHM score (participants with lower CHM scores had stronger negative associations between the log of TEM structure counts and platelets than participants with higher CHM scores; Table 7).

**Table 7:** Results of multiple linear regression modeling of complete blood count components

	CNT/F metric β estimate (*p*-value)						
	ln(Leukocytes)^a^	ln(Neutrophils)^b^	ln(Lymphocytes)^c^	ln(Monocytes)^d^	ln(Platelets)^e^	Hemoglobin^2f^	Hematocrit^2g^
*Exposure variable – untransformed*							
EC–inhalable (μg/m^3^)	-2.17E-4 (0.692)	-9.34E-5 (0.899)	-3.74E-4 (0.577)	-5.94E-3 (0.364)	2.09E-5 (0.965)	-1.80E-2 (0.775)	-0.237 (0.618)
EC-respirable (μg/m^3^)	-3.92E-3 (0.468)	-3.78E-3 (0.533)	-3.32E-3 (0.556)	-4.39E-3 (0.428)	1.59E-3 (0.692)	-0.294 (0.578)	-2.33 (0.563)
TEM-total (structures/cm^3^)	-8.30E-2 (0.080)^h^	-9.06E-2 (0.154)^h^	-7.51E-2 (0.201)^h^	-3.03E-2 (0.612)	-3.76E-3 (0.929)	-4.08 (0.463)	-26.9 (0.533)
CNT/F found in sputum	5.75E-2 (0.380)	8.21E-2 (0.355)	1.90E-2 (0.980)	4.79E-2 (0.542)	-8.40E-2 (0.109)	2.17 (0.332)	32.4 (0.566)
CNT/F emp. duration (years)	-5.78E-3 (0.316)	-7.34E-2 (0.340)	1.70E-3 (0.814)	3.78E-3 (0.594)	-1.27E-3 (0.804)	0.400 (0.548)	5.28 (0.308)
*Exposure variable – log-transformed*							
ln(EC-inhalable)	4.05E-3 (0.592)	8.15E-3 (0.419)	-4.31E-3 (0.646)	1.34E-2 (0.149)^h^	-3.74E-3 (0.575)	1.44 (0.103)	10.3 (0.127)
ln(EC-respirable)	-1.06E-2 (0.373)	-1.43E-2 (0.371)	-2.40E-3 (0.869)	-9.98E-3 (0.494)	1.30E-2 (0.206)	-0.460 (0.738)	6.02E-3 (0.999)
ln(TEM-total)	-1.28E-3 (0.885)	6.89E-3 (0.560)	-1.14E-2 (0.303)	-3.20E-3 (0.771)	-1.28E-2 (0.101)^h^	1.82 (0.0788)^h^	15.9 (0.0433)^h^
*Effect modification* ^*i*^							
Male	-8.24E-2	-9.31E-2	-7.85E-2	1.45E-2	-6.60E-3	1.85	18.5
Female	-9.14E-2	-5.31E-2	-2.50E-2	-5.96E-3	-3.59E-2	1.74	7.51
*p* for interaction	0.9596	0.867	0.805	0.531	0.001	0.965	0.553
White race, non-Hispanic	-8.29E-2	-9.27E-2	-7.52E-2	6.66E-4	-1.23E-2	1.86	14.8
All other races and Hispanic	-6.23	-6.54	-4.44	1.46E-2	-2.02E-2	1.33	34.0
*p* for interaction	0.009	0.041	0.124	0.646	0.803	0.898	0.542
Age <40	-9.04E-2	-0.111	-6.72E-2	5.27E-3	-2.19E-2	1.50	16.0
Age ≥40	1.39E-2	0.178	-2.17E-1	1.76E-2	-8.10E-3	1.95	15.9
*p* for interaction	0.529	0.191	0.478	0.461	0.067	0.649	0.995
Education < college degree	-8.91E-2	-1.06E-1	-1.18E-1	1.64E-2	-1.64E-2	0.530	4.27
Education ≥ college degree	-7.65E-2	-7.45E-2	-2.81E-2	1.23E-2	-1.11E-2	2.55	22.7
*p* for interaction	0.890	0.796	0.427	0.836	0.513	0.056	0.020
No lung disease^j^	-8.87E-2	-1.18E-1	-1.19E-2	1.41E-2	-5.11E-3	1.72	14.0
Has lung disease	-7.96E-2	-6.86E-2	-1.11E-1	1.27E-2	-1.58E-2	1.85	16.5
*p* for interaction	0.924	0.733	0.388	0.934	0.170	0.900	0.747
CHM score <5	-8.85E-2	-9.98E-2	-8.42E-2	1.45E-2	-2.20E-2	2.25	17.4
CHM score ≥5	2.86E-2	9.34E-2	6.90E-2	1.28E-2	-8.79E-3	1.42	14.5
*p* for interaction	0.547	0.458	0.533	0.920	0.085	0.688	0.856

Abbreviations—*CNT/F* carbon nanotubes or nanofibers, *CHM* cardiovascular health metric, *EC* elemental carbon, *TEM* transmission electron microscopy

^a^Leukocytes adjusted for total U/FP counts per cm^3^ and self-reported current dust exposure

^b^Neutrophils adjusted for total U/FP counts per cm^3^ and self-reported current dust exposure

^c^Lymphocytes adjusted for NSAID use, CHM score, and self-reported past exposure to nanomaterials other than CNT/F

^d^Monocytes adjusted for race/ethnicity, current respiratory infection, total U/FP counts per cm^3^ and self-reported past polymer exposure

^e^Platelets adjusted for race/ethnicity

^f^Hemoglobin adjusted for sex, race/ethnicity, and CHM score

^g^Hematocrit adjusted for sex, race/ethnicity, CHM score, and current respiratory infection

^h^CNT/F exposure metric associated with the lowest Schwarz’s Bayesian Criterion value (best fit)

^i^Parameter estimates and p-values are shown for the CNT/F exposure metric identified as having the best fit

^j^Self-reported respiratory allergy, asthma, or chronic obstructive pulmonary disease

### Regression model diagnostics

Model residuals for all outcomes were reasonably normally distributed, and no apparent patterns were observed between residuals and predictors. For RHR, a highly influential observation was detected. Removal of this observation decreased the association between (untransformed) inhalable EC and heart rate, but the log-transformed inhalable EC remained significantly associated with heart rate, and the pattern of effect modification with sex remained.

## Discussion

**Fig. 2:**
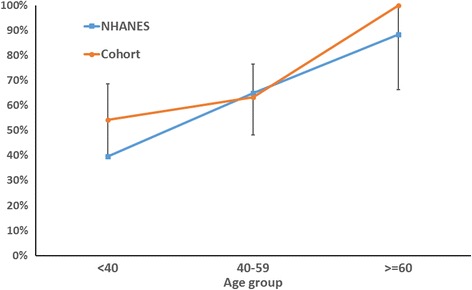
Percent of population hypertensive or pre-hypertensive. Abbreviations: CNT/F – carbon nanotubes and nanofiber; NHANES – National Health and Nutrition Examination Survey. NHANES data from NHANES 2013. 95% confidence intervals were estimated for the CNT/F workers, assuming an exact binomial distribution

Since the commercialization of carbon nanotubes and nanofibers over the past 10-15 years, concern has been raised about their possible human health effects, due to their pulmonary and cardiovascular toxicity in animal models and through analogy with fibrous structures such as asbestos [3, 42]. In this study, we observed little evidence of associations between different metrics of CNT/F exposure and clinically relevant outcomes, including pulmonary function, BP, and most hematologic elements, among 108 U.S. workers exposed routinely to CNT/F. Few workers showed evident pulmonary function decrement (below the lower limit of normal) for the age, sex, race and height-adjusted population, and no negative associations were found between these metrics and sub-clinical pulmonary function levels. We found that a relatively high percentage of the CNT/F workers were pre- or hypertensive: when including those who self-reported a physician diagnosis of hypertension, the percentage rose from 58% to 62%. However, the percentage by age group was similar to that in the general U.S. population (Fig. 2), as reported in NHANES [33], and BP was not associated with CNT/F exposure.

We identified a few health measures significantly associated with some metrics of CNT/F exposure: 14% of workers developed respiratory allergy after starting work with CNT/F, and the odds of reporting respiratory allergies increased with length of time spent working with CNT/F. Both respiratory allergy development and RHR were significantly positively associated with inhalable EC concentration. Respirable EC concentration was positively associated with RHR, whereas length of time spent working with CNT/F was negatively associated with RHR. While the overall reporting of any chest symptom (49%) and respiratory allergy (54%) was relatively high in our study, such participants did not exhibit greater sensitivity to CNT/F for the pulmonary, cardiovascular, or hematologic metrics we evaluated. The negative association between total leukocyte and total TEM structure concentrations was stronger for Hispanic and non-white workers than for white workers. Hemoglobin concentration and hematocrit percentage were positively associated with CNT/F structure count concentrations (log-transformed), particularly among workers with higher education levels, and negative associations between the same metric and platelet concentrations were significantly stronger among female workers, younger workers, and workers with worse cardiovascular health.

There have been few studies of clinically relevant health metrics among workers exposed to CNT/F. A small study in Korea of nine workers manufacturing MWCNT [11] found none had depressed (<80% predicted) FVC or FEV1. Two workers exhibited abnormal monocyte counts, and several others showed abnormal levels of certain hepatic enzymes. The Korean workers had inhalable EC exposure levels in a range about ten times higher than the median level observed in our study. Liou et al. [10] measured pulmonary function among a group of 227 workers handling a variety of engineered nanomaterials (23% were exposed only to CNT), compared to a group of unexposed workers. No association was found between exposure and lung function metrics. That study team did observe evidence of respiratory and dermal symptoms (sneezing and dermatitis) in the more highly exposed workers in this population [39]. No association was observed between CNT/F exposure and pulmonary function in a small European study [12].

No human studies, to our knowledge, have evaluated the association of CNT/F exposure with RHR or resting BP. RHR, measured cross-sectionally, was associated strongly with mortality in a healthy male population: risk of death in a 16-year period rose 16% (95% confidence interval: 10-22%) per 10 beat-per-minute heart rate level, after adjusting for physical fitness and other covariates [17]. Therefore, it is important to identify environmental or occupational contributors to high RHR. We consider our finding of an association between RHR and inhalable EC to be preliminary until it is replicated in other groups, particularly because a study of MWCNT exposure in spontaneously hypertensive rats found a depression in RHR [9]. The positive association we observed between CNT/F exposure and hematocrit could reflect less hydration among CNT/F-exposed workers, as the tasks associated with this work can involve heat exposure.

It is important to note that very few workers (n=7) had background-corrected, respirable EC exposure concentrations above the NIOSH REL of 1 μg/m^3^ [13]. However, a much larger percentage had inhalable concentrations above this level (although there is no REL established for the inhalable EC air concentration). Therefore, it is of considerable interest whether the inhalable or respirable fraction of EC is a more health-relevant metric. We found that inhalable EC or total structure count concentration tended to relate more strongly to the few pulmonary, cardiovascular, or hematologic endpoints that were associated with a CNT/F metric. This finding is similar to that observed for blood and sputum biomarkers of early effect in the same study group [23], and an *in vivo* study of MWCNT that primarily deposited in the conducting airways [43]. These findings suggest that protective standards should also consider aerosol size fractions larger than respirable, such as the inhalable or thoracic fractions.

No outcomes were significantly associated with the presence of CNT/F in sputum, and this metric showed little association with biomarker levels in blood or sputum [23]. The sensitivity of this metric is probably low, given the poor quality of the sputum (which indicates that much of it may have originated in the oral cavity) and the small percentage (<1%) of the specimen that was inspected using the dark-field microscopy method. It is notable that, in contrast to the air sampling results [24, 26] all CNT/F structures observed in sputum were single fibers (e.g., Fig. 1), suggesting that some dissociation of agglomerates may occur after oral cavity or respiratory tract intake.

We observed several other occupational exposures to be associated with some respiratory, cardiovascular, and hematologic metrics: polymers, solvents and strong acids were the most commonly self-reported. Measured U/FP concentrations in the workplace were more important predictors of blood pressure and leukocyte components than CNT/F exposure. The ambient air quality metric most consistently associated with health measures was the U/FP count concentration, measured with a CPC. While U/FP concentration was only weakly correlated with CNT/F exposure, it was significantly positively associated with systolic BP and leukocyte, neutrophil, and lymphocyte counts. (Additional file 1: Tables S8 and S9). This finding, if indicative of a causal association, could have utility for the health of the workers, as it confirms the importance of minimizing exposure to U/FP in the workplace, aside from specific concern about CNT/F. U/FP are common in workplaces and can be caused by a variety of combustion sources such as vehicle exhaust and reactor byproducts, as well as industrial dryers, compressors, and vacuum cleaners. The CPC is a relatively inexpensive, easy-to-use instrument for measuring real-time concentrations of 10-1000 nm-diameter particulates in the workplace.

Strengths of this study include the consistent exposure and outcome assessment methodologies, the use of a wide range of CNT/F exposure metrics, and the large number of facilities included, with a nationally representative range of CNT/F types. This study is subject to a number of important limitations. We were unable to verify the accuracy of self-reported chest symptoms and respiratory diseases. The cross-sectional design hampers interpretability of temporality of any observed associations and is subject to selection bias, in that more susceptible workers may have dropped out of the CNT/F workforce. The positive association between CNT/F duration and lung function suggests such selection could exist in this workforce. The design also limits the ability to estimate respiratory symptom or allergy development, given the potential correlation of these events with follow-up time (although we found that age was not related to risk of development of symptoms or allergies). The small study size and generally low exposure levels limit the statistical power to detect subclinical effects on the outcomes. This limitation might be reduced through pooling of these data with similarly conducted studies worldwide (e.g., [12]). Variability in the types of CNT/F included in this study presented a challenge, as the toxic potential of carbon nanotubes varies substantially by material characteristics [44]. Most facilities in our study were using CNTs of a “tangled” morphology, not the rigid form deemed to be “possibly carcinogenic” [45]. Given the very recent commercialization of CNT/F, there is short latency for developing restrictive or obstructive lung disease: the average duration of time these participants worked with CNT/F was 4 years. The exposure metrics did not account for use of protective equipment, such as respirators, as we could not assess the efficacy of use on an individual basis. It is uncertain to what degree our cross-sectionally measured air concentrations of EC or CNT/F structures accurately reflect past exposure for workers in our study. The mean and median exposures measured here were lower than in previous studies of the same workforce (e.g., in [24]). However, this likely was influenced by differences in participant selection for these studies: 37% of participants in this study reported not directly handling CNT/F, whereas our previous studies [24] focused on workers directly handling these materials. Lastly, the representativeness of the facilities included in this study is uncertain, as only 1/3 responded to our initial request for information about their use of CNT/F. However, our study targeted companies with the highest volume of CNT/F handling in the U.S., and the overall non-selective and high employee participation rate suggests that within-facility representativeness was high.

## Conclusions

We found that most pulmonary, cardiovascular and hematologic measures were unrelated to metrics of CNT/F exposure in U.S. workplaces. Respiratory allergy development and resting heart rate were positively associated with inhalable concentrations of EC. Hemoglobin and hematocrit concentrations were positively related to CNT/F structure count concentrations. These findings require confirmation in other exposed populations, preferably with longitudinal designs. The low exposure levels and lack of substantive clinical impairment suggest that the CNT/F industry is responsibly handling these materials, and efforts should continue to use a comprehensive approach to minimize exposure to CNT/F, fine particulates and other potentially hazardous agents in these workplaces.

## Additional files

Additional file 1: **Table S1.** Participation rates by facility. **Table S2.** Current and past self-reported exposure frequency among cross-sectional study participants. **Table S3.** Scoring method for risk factors used in cardiovascular health metrics score. **Table S4.** Distribution of cardiovascular health metric (CHM) score values, where a higher score implies better cardiovascular health. **Table S5.** Frequency of chest symptoms or respiratory illnesses among 108 study participants. **Table S6.** Results of univariable logistic regression modeling of personal characteristics and occupational exposures for development of chest symptoms or respiratory allergy after the start of CNT/F work**. Table S7.** Results of univariable linear regression modeling of pulmonary function metrics (highlight indicates selected in “best model” by Schwarz Bayesian Criterion and considered as potential confounder in multiple linear regression model with main exposure variables). **Table S8.** Results of univariable linear regression modeling of cardiovascular metrics (highlight indicates selected in “best model” by Schwarz Bayesian Criterion and considered as potential confounder in multiple linear regression model with main exposure variables). **Table S9.** Results of univariable linear regression modeling of natural log (ln)-transformed WBC and differential metrics (highlight indicates selected in “best model” by Schwarz Bayesian Criterion and considered as potential confounder in multiple linear regression model with main exposure variables). **Table S10.** Results of univariable linear regression modeling of other transformed CBC metrics (highlight indicates selected in “best model” by Schwarz Bayesian Criterion and considered as potential confounder in multiple linear regression model with main exposure variables). (DOCX 41 kb)

Additional file 2: Questionnaire. (DOCX 37 kb)

Additional file 3: Supplementary information. (DOCX 24 kb)
