# Supplementary material for: Association of pulmonary, cardiovascular, and hematologic metrics with carbon nanotube and nanofiber exposure among U.S. workers: a cross-sectional study
Source: Part Fibre Toxicol. 2018 May 16;15:22. doi: 10.1186/s12989-018-0258-0 (PMC5956815; doi:10.1186/s12989-018-0258-0)
Supplement: Supplementary file 1 — Table S1. Participation rates by facility. Table S2. Current and past self-reported exposure frequency among cross-sectional study participants. Table S3. Scoring method for risk factors used in cardiovascular health metrics score. Table S4. Distribution of cardiovascular health metric (CHM) score values, where a higher score implies better cardiovascular health. Table S5. Frequency of chest symptoms or respiratory illnesses among 108 study participants. Table S6. Results of univariable logistic regression modeling of personal characteristics and occupational exposures for development of chest symptoms or respiratory allergy after the start of CNT/F work. Table S7. Results of univariable linear regression modeling of pulmonary function metrics (highlight indicates selected in “best model” by Schwarz Bayesian Criterion and considered as potential confounder in multiple linear regression model with main exposure variables). Table S8. Results of univariable linear regression modeling of cardiovascular metrics (highlight indicates selected in “best model” by Schwarz Bayesian Criterion and considered as potential confounder in multiple linear regression model with main exposure variables). Table S9. Results of univariable linear regression modeling of natural log (ln)-transformed WBC and differential metrics (highlight indicates selected in “best model” by Schwarz Bayesian Criterion and considered as potential confounder in multiple linear regression model with main exposure variables). Table S10. Results of univariable linear regression modeling of other transformed CBC metrics (highlight indicates selected in “best model” by Schwarz Bayesian Criterion and considered as potential confounder in multiple linear regression model with main exposure variables). (DOCX 41 kb) [file 12989_2018_258_MOESM1_ESM.docx]

**Additional File 1 Tables**

Association of pulmonary, cardiovascular, and hematologic metrics with carbon nanotube and nanofiber exposure among U.S. workers: A cross-sectional study

Mary K. Schubauer-Berigan, Matthew M. Dahm, Aaron Erdely, John D. Beard, M. Eileen Birch, Douglas E. Evans, Joseph E. Fernback, Robert R. Mercer, Stephen J. Bertke, Tracy Eye, Marie A. de Perio

**Table of Contents**

Table S1…………………………………………………………….………………………………... 2

Table S2…………………………………………….………………………………………………... 2

Table S3…………………………………………………….………………………………………... 3

Table S4………………………………………………………………….…………………………... 3

Table S5……………………………….……………………………………………………………... 4

Table S6………………………….…………………………………………………………………... 5

Table S7………………………………………………….…………………………………………. 6

Table S8………………………………………………….…………………………………………. 7

Table S9………………………………………………….…………………………………………. 8

Table S10…………………………………………………………………………………………. 9

**Table S1. Participation rates by facility**

| **Facility number** | **Type of ECN made or used** | **N total workers at facility** | **N in ECN unit** | **N enrolled (% of N in ECN unit)** |
| --- | --- | --- | --- | --- |
| 1 | MWCNT | 3 | 3 | 3 (100%) |
| 2 | CNF, MWCNT | 5 | 4 | 4 (100%) |
| 3 | CNF, MWCNT | 6 | 6 | 3 (50%) |
| 4 | MWCNT | 6 | 6 | 6 (100%) |
| 5 | MWCNT, SWCNT | 12 | 7 | 6 (85.7%) |
| 6 | MWCNT | 500 | 7 | 7 (100%) |
| 7 | MWCNT | 11 | 11 | 11 (100%) |
| 8 | MWCNT | 12 | 12 | 10 (83.3%) |
| 9 | MWCNT, SWCNT | 14 | 12 | 11 (91.7%) |
| 10 | MWCNT, SWCNT | 21 | 16 | 12 (75%) |
| 11 | MWCNT | 25 | 20 | 16 (80%) |
| 12 | MWCNT, SWCNT | 60 | 40 | 19 (47.5%) |
|  | -- | 675 | 144 | 108 (75%) |

Abbreviations: CNF – carbon nanofibers; ECN – engineered carbonaceous nanomaterials; MWCNT – multi-walled carbon nanotubes; SWCNT – single-walled carbon nanotubes

**Table S2. Current and past self-reported exposure frequency among cross-sectional study participants**

| **Chemical exposure type** | **Currently exposed** | **Past exposed** | **Clinical outcomes in which evaluated as a potential confounder** |
| --- | --- | --- | --- |
| Solvents | 52% | 47% | All |
| Polymers | 27% | 27% | All |
| Strong acids | 21% | 22% | Lung function metrics, chest symptoms |
| “Other nano” (not CNT/F) | 15% | 13% | All |
| “Other dust” (not smoke or Si) | 13% | 43% | All |
| Strong bases | 6% | 6% | None (too-low percentage, not strong risk factors) |
| Silica | 5% | 11% | All (combined with “other dust”) |
| Carbon powder | 5% | 7% | All (combined with “other dust”) |
| Diesel | 1% | 10% | All (combined with “other dust) |
| Paint | 1% | 8% | None |
| Fumes | 1% | 7% | None (too-low percentage) |
| Lead | 0% | 9% | None (too-low percentage) |
| Metalworking | 0% | 7% | None (too-low percentage, not strong risk factors) |

Abbreviations: CNT/F – carbon nanotubes and nanofibers; Si - silica

**Table S3. Scoring method for risk factors used in cardiovascular health metrics score**

| **Cardiovascular risk factor** | **Value associated with score=0** | **Value associated with score=1** | **N missing** | **% with score=1** |  |
| --- | --- | --- | --- | --- | --- |
| Body mass index | ≥25 kg/m^2^ | <25 kg/m^2^ | 2 | 34.0% | |
| Waist circumference | ≥102 cm (men)  ≥88 cm (women) | <102 cm (men)  <88 cm (women) | 2 | 69.8% | |
| Hypertension diagnosis | Yes | No | 0 | 72.2% | |
| Diabetes diagnosis | Yes | No | 0 | 94.4% | |
| Current cigarette smoker | Yes^1^ | No | 0 | 84.3% | |
| Using cholesterol-lowering medication | Yes | No | 0 | 87.0% | |

^1^Includes one person who quit within past eight months

**Table S4. Distribution of cardiovascular health metric (CHM) score values, where a higher score implies better cardiovascular health.**

| **CHM score** | **N with score** | **Percent of non-missing^§^** | **Cumulative percentage** |
| --- | --- | --- | --- |
| 0-2 | 11 | 10.4% | 10.4% |
| 3 | 11 | 10.4% | 20.8% |
| 4 | 26 | 24.5% | 45.3% |
| 5 | 33 | 31.1% | 76.4% |
| 6 | 25 | 23.6% | 100% |

^§^CHM score is missing for two participants

**Table S5. Frequency of chest symptoms or respiratory illnesses among 108 study participants.**

| Chest symptom or respiratory illness outcome | N (%) reporting outcome before start of CNT/F-work | N (%) outcome-free before start of CNT/F-work | N (% of initially outcome-free) reporting outcome after start of nano-work |
| --- | --- | --- | --- |
| Chest symptom (usual cough, phlegm or wheeze) | 45 (42%) | 63 (58%) | 13 (21%) |
| Respiratory allergy | 44 (41%) | 64 (59%) | 9* (14%) |
| Asthma | 9 (8%) | 99 (92%) | 0 |
| COPD (incl. chronic bronchitis and emphysema) | 6 (6%) | 102 (94%) | 0 |

Abbreviations: CNT/F – carbon nanotubes and nanofibers; COPD – chronic obstructive pulmonary disease

*Only 1 case also reported chest symptom initiation after start of CNT/F work

**Table S6. Results of univariable logistic regression modeling of personal characteristics and occupational exposures for development of chest symptoms or respiratory allergy after the start of CNT/F work**

|  | **Odds ratio (OR) (p-value)** | |
| --- | --- | --- |
|  | **Chest symptom** | **Respiratory allergy** |
|  |  |  |
| Age (OR per 10 year) | 1.04 (0.87) | 1.02 (0.94) |
| Sex (M:F) | 1.05 (0.94) | 0.39 (0.26) |
| Race (White, non-Hisp: all others) | 1.37 (0.71) | NC^a^ |
| Cigarette pack-years (OR at 10 pack years) | 1.60 (0.077) | 0.99 (0.96) |
| CHM score (high:low) | 0.35 (0.098) | 1.24 (0.78) |
| Pneumonia<age16 | 2.20 (0.33) | 1.68 (0.57) |
| Solvent (OR exposed: unexposed) – current  –past | 2.84 (0.12)  1.26 (0.71) | 2.23 (0.28)  0.96 (0.96) |
| Strong acid (OR exposed: unexposed) – current  –past | 2.50 (0.18)  3.04 (0.092) | 0.92 (0.93)  0.56 (0.59) |
| Polymers (OR exposed: unexposed)– current  –past | 5.67 (0.0076)  1.61 (0.47) | 2.87 (0.17)  0.92 (0.93) |
| Other Dust (OR exposed: unexposed) –current  –past | 0.33 (0.26)  1.49 (0.53) | 1.62 (0.54)  0.48 (0.32) |
| Other nano (OR exposed: unexposed) – current  –past | 2.03 (0.33)  0.96 (0.96) | 1.96 (0.47)  0.86 (0.89) |

Abbreviations— CHM: cardiovascular health metric; CNT/F: carbon nanotubes or nanofibers; F: female; M: male; W: white

^a^NC: not calculable. All cases were among White, non-Hispanic participants.

**Table S7. Results of univariable linear regression modeling of pulmonary function metrics (highlight indicates selected in “best model” by Schwartz Bayesian Criterion and considered as potential confounder in multiple linear regression model with main exposure variables)**

|  | **β estimate (p-value)** | | | |
| --- | --- | --- | --- | --- |
|  | **FVC PP** | **FEV1/FVC PP** | **ln(FEF25-75 PP)** | **PEF PP** |
| ***Exposure variable*** | | | | |
| EC–inhalable  ln(EC–inhalable) | -2.16E-4 (0.425)  6.71E-4 (0.855) | 2.53E-4 (0.155)  1.27E-3 (0.598) | 1.02E-3 (0.185)  7.33E-3 (0.479) | 3.97E-5 (0.908)  -5.10E-3 (0.271) |
| EC-respirable  ln(EC-respirable) | -1.06E-3 (0.640)  6.56E-3 (0.214) | 1.87E-3 (0.208)  6.28E-4 (0.857) | 8.65E-3 (0.175)  0.0143 (0.340) | 1.75E-4 (0.951)  -9.19E-3 (0.169) |
| TEM-total mean  ln(TEM-total mean) | 1.94E-2 (0.417)  6.82E-3 (0.117) | -4.80E-4 (0.976)  2.55E-3 (0.376) | 0.0469 (0.489)  0.0157 (0.204) | -0.0164 (0.588)  3.40E-3 (0.540) |
| Sputum presence | 3.89E-3 (0.901) | -5.15E-3 (0.804) | -0.0404 (0.648) | 0.0121 (0.770) |
| CNT/F duration emp | 6.76E-4 (0.811) | 2.83E-3 (0.125) | 0.0141 (0.076) | 4.49E-3 (0.207) |
|  |  |  |  |  |
| ***Confounder variable – Personal characteristics*** | | | | |
| Age | -8.50E-5 (0.922) | 2.76E-4 (0.643) | -4.61E-4 (0.857) | -3.81E-5 (0.973) |
| Sex (F:M) | 2.89E-3 (0.916) | 5.97E-3 (0.750) | 0.0578 (0.475) | 0.399 (0.269) |
| Race (W,non-Hisp:Oth) | 0.0913 (0.0046) | -0.0157 (0.467) | 0.049 (0.596) | -9.62E-3 (0.817) |
| Cigarette pack-years | -3.85E-4 (0.666) | -1.22E-3 (0.036) | -6.57E-3 (0.0082) | -1.37E-3 (0.225) |
| CHM score (high:low) | 0.0242 (0.289) | 6.04E-3 (0.689) | 0.0538 (0.406) | 0.0162 (0.578) |
| Pneumonia<age16 | -0.0459 (0.180) | -- | -- | -- |
| Current asthma | -- | -0.0427 (0.105) | -0.165 (0.146) | -0.0380 (0.458) |
| Current COPD | -- | 0.0286 (0.412) | 0.0470 (0.754) | 8.27E-3 (0.902) |
| Current cold | 6.45E-3 (0.873) | 6.70E-3 (0.801) | -0.0270 (0.813) | -0.0516 (0.312) |
| ***Confounder variable – Workplace exposures*** | | | | |
| Mean U/FP counts | -5.57E-9 (0.993) | 1.15E-7 (0.797) | 1.10E-8 (0.995) | 1.68E-7 (0.845) |
| Mean UP counts | 7.32E-7 (0.380) | 2.19E-7 (0.691) | 1.24E-6 (0.599) | 9.26E-7 (0.382) |
| Mean PM-2.5 mass | 1.51E-3 (0.262) | -7.44E-4 (0.403) | -2.51E-3 (0.513) | -8.76E-4 (0.610) |
| Solvent - current  --past | -0.0113 (0.619)  -0.0613 (0.0062) | -0.0156 (0.296)  -5.68E-4 (0.970) | -0.0929 (0.148)  -0.0875 (0.173) | -8.14E-3 (0.778)  -0.0467 (0.104) |
| Strong acid – current  --past | 0.0764 (0.0044)  0.0323 (0.236) | -0.0325 (0.0688)  -0.0274 (0.127) | -0.0406 (0.601)  -0.0576 (0.457) | 0.0345 (0.383)  -0.0293 (0.399) |
| Polymers – current  --past | -4.54E-3 (0.859)  0.0101 (0.691) | 0.0133 (0.431)  0.0215 (0.196) | 0.0368 (0.611)  0.0954 (0.182) | -5.90E-3 (0.856)  -0.0200 (0.534) |
| Dust –current  --past | -0.0333 (0.222)  8.10E-3 (0.722) | 0.0209 (0.243)  -0.0166 (0.268) | 0.0239 (0.758)  -0.0702 (0.276) | 0.0259 (0.454)  -0.0617 (0.031) |
| Other nano – current  --past | -0.0562 (0.0714)  0.0178 (0.591) | 0.0217 (0.295)  -0.0190 (0.385) | 0.0167 (0.851)  -0.0703 (0.455) | 0.0595 (0.134)  -0.0731 (0.0807) |

Abbreviations— CHM: cardiovascular health metric; CNT/F: carbon nanotubes or nanofibers; COPD: chronic obstructive pulmonary disease; EC: elemental carbon; F: female; FEV1: forced expiratory volume in one second; FVC: forced vital capacity; FEF25-75: forced expiratory fraction between 25 and 75% of maximal; M: male; PEF: peak expiratory flow; PM: particulate matter; PP: percent predicted; TEM: transmission electron microscopy; U/FP: ultrafine and fine particulate; UP: ultrafine particulate; W: white

**Table S8. Results of univariable linear regression modeling of cardiovascular metrics (highlight indicates selected in “best model” by Schwartz Bayesian Criterion and considered as potential confounder in multiple linear regression model with main exposure variables)**

|  | **β estimate (p)** | | |
| --- | --- | --- | --- |
|  | **Systolic BP** | **Diastolic BP** | **Heart rate** |
| EC–inhalable  ln(EC–inhalable) | -0.0139 (0.6782)  0.160 (0.7218) | -0.0183 (0.4425)  0.189 (0.5542) | 0.0319 (0.2252)  0.853 (0.0146) |
| EC-respirable  ln(EC-respirable) | -0.140 (0.6143)  -0.114 (0.8610) | -0.108 (0.5863)  0.0306 (0.9473) | 0.193 (0.3787)  0.164 (0.7482) |
| TEM-total mean  ln(TEM-total mean) | -0.027 (0.9927)  0.527 (0.3180) | 0.267 (0.8985)  0.354 (0.3468) | -0.0333 (0.9885)  0.304 (0.4656) |
| Sputum presence | 2.07 (0.5971) | 0.434 (0.8799) | 0.601 (0.8542) |
| CNT/F duration emp | 0.512 (0.1353) | 0.0788 (0.7486) | -0.734 (0.0061) |
| ***Confounder variable – Personal characteristics*** | | | |
| Age | 0.310 (0.0029) | 0.0817 (0.2918) | -0.119 (0.1604) |
| Sex (F:M) | -8.12 (0.0108) | -3.41 (0.1546) | 5.74 (0.0285) |
| Race (W,non-Hisp:Oth) | 2.34 (0.5319) | 0.537 (0.8498) | -0.0449 (0.9884) |
| Cigarette pack-years | 0.292 (0.0067) | 0.162 (0.0361) | 0.123 (0.1532) |
| CHM score (high:low) | -6.69 (0.0147) | -5.69 (0.0034) | -5.34 (0.0136) |
| Alcohol-drinks per week | 0.0146 (0.5929) | 0.111 (0.5686) | 0.0560 (0.7954) |
| ***Confounder variable – Workplace exposures*** | | | |
| Mean U/FP counts | 2.00E-4 (0.0148) | 6.50E-5 (0.272) | 5.21E-5 (0.4270) |
| Mean UP counts | 2.32E-4 (0.0223) | 6.72E-5 (0.3590) | 4.56E-5 (0.5746) |
| Mean PM-2.5 mass | 0.310 (0.0544) | 0.0600 (0.6040) | -0.163 (0.2018) |
| Solvent – current  –past | 3.29 (0.2332)  2.11 (0.4459) | 0.724 (0.7141)  0.939 (0.6343) | -3.30 (0.1298)  0.311 (0.8869) |
| Strong acid – current  –past | 3.84 (0.2516)  -2.32 (0.4822) | 4.13 (0.0827)  -3.00 (0.2013) | -0.442 (0.8676)  -1.18 (0.6525) |
| Polymers – current  –past | 0.612 (0.8440)  0.968 (0.7554) | -0.736 (0.7395)  2.06 (0.3501) | -4.68 (0.0540)  -3.21 (0.1885) |
| Dust –current  –past | 4.68 (0.1554)  1.39 (0.6164) | 1.55 (0.5110)  0.745 (0.7057) | -1.12 (0.6675)  1.02 (0.6411) |
| Other nano – current  –past | 2.45 (0.5267)  -2.98 (0.4664) | 2.75 (0.6756)  2.12 (0.4664) | -2.42 (0.4273)  -5.90 (0.0656) |

Abbreviations—BP: blood pressure; CHM: cardiovascular health metric; CNT/F: carbon nanotubes or nanofibers; EC: elemental carbon; F: female PM: particulate matter; TEM: transmission electron microscopy; U/FP: ultrafine and fine particulate; UP: ultrafine particulate; W: white

**Table S9. Results of univariable linear regression modeling of natural log (ln)-transformed WBC and differential metrics (highlight indicates selected in “best model” by Schwartz Bayesian Criterion and considered as potential confounder in multiple linear regression model with main exposure variables)**

|  | **β estimate (*p*)** | | | |
| --- | --- | --- | --- | --- |
| **Transformed metric🡪** | **ln-Leukocytes** | **ln-Neutrophils** | **ln-Lymphocytes** | **ln-Monocytes** |
| EC–inhalable  ln(EC–inhalable) | -4.22E-4 (0.4622)  6.73E-3 (0.3980) | -3.43E-4 (0.6486)  0.0111 (0.2890) | -3.44E-4 (0.6113)  1.40E-3 (0.8817) | -5.29E-4 (0.4742)  8.57E-3 (0.4031) |
| EC-respirable  ln(EC-respirable) | -4.57E-3 (0.3398)  -6.47E-3 (0.6016) | -4.63E-3 (0.4610)  -9.85E-3 (0.5443) | -2.78E-3 (0.6221)  7.75E-4 (0.9577) | -5.41E-3 (0.3800)  -0.0136 (0.3953) |
| TEM-total mean  ln(TEM-total mean) | -0.0637 (0.2057)  2.79E-3 (0.7654) | -0.0687 (0.2984)  0.0114 (0.3502) | -0.0556 (0.3498)  -7.81E-3 (0.4770) | -0.0656 (0.3123)  -5.64E-3 (0.6390) |
| Sputum presence | 0.0362 (0.5941) | 0.0558 (0.5389) | -0.0154 (0.8409) | 0.0588 (0.5166) |
| CNT/F duration emp | -6.32E-3 (0.2993) | -8.05E-3 (0.3128) | -1.84E-3 (0.7983) | 2.48E-4 (0.9748) |
| ***Confounder variable – Personal characteristics*** | | | |  |
| Age | -6.94E-4 (0.7159) | 7.52E-4 (0.7635) | -3.44E-3 (0.1359) | -7.25E-4 (0.7683) |
| Sex (F:M) | 0.113 (0.0664) | 0.173 (0.0319) | 0.0704 (0.3376) | -0.0148 (0.8505) |
| Race (W,non-Hisp:Oth) | 0.155 (0.0342) | 0.159 (0.0952) | 0.0923 (0.2886) | 0.248 (0.0089) |
| Cigarette pack-years | 1.53E-3 (0.4214) | 8.64E-4 (0.7290) | 2.50E-3 (0.2639) | 2.78E-3 (0.2548) |
| CHM score (high:low) | -0.103 (0.0364) | -0.110 (0.0876) | -0.110 (0.0571) | -0.0405 (0.5252) |
| Alcohol-drinks per week | 8.65E-4 (0.8583) | 2.89E-4 (0.9636) | -9.66E-4 (0.8657) | 5.43E-3 (0.3828) |
| Current resp infection | -0.144 (0.1079) | -0.134 (0.2572) | -0.0848 (0.4251) | -0.431 (0.0001) |
| Current NSAID use | -0.0106 (0.8503) | 0.0385 (0.5997) | -0.105 (0.1089) | 0.0515 (0.4752) |
| Anticholesterol med use | 0.0703 (0.3342) | 0.134 (0.1581) | -0.0215 (0.8027) | 0.0113 (0.9043) |
| Antihypertensive use | -8.53E-3 (0.9012) | 0.0103 (0.9090) | -0.0142 (0.8610) | -0.0872 (0.3231) |
| ***Confounder variable – Workplace exposures*** | | | |  |
| Mean U/FP counts | 3.81E-6 (0.0072) | 4.16E-6 (0.0258) | 4.02E-6 (0.0165) | 2.95E-6 (0.1103) |
| Mean UP counts | 3.15E-6 (0.0752) | 3.45E-6 (0.1376) | 3.12E-6 (0.1360) | 3.69E-6 (0.1061) |
| Mean PM-2.5 mass | 8.15E-4 (0.7728) | -6.56E-4 (0.8592) | 3.42E-3 (0.3029) | -4.45E-4 (0.9026) |
| Solvent – current  –past | -0.0559 (0.2580)  -0.0976 (0.0462) | -0.100 (0.1212)  -0.138 (0.0314) | 5.47E-3 (0.9254)  0.0600 (0.3021) | 0.0150 (0.8142)  -0.0519 (0.4146) |
| Polymers – current  –past | 5.33E-3 (0.9234)  -0.0611 (0.2626) | -9.62E-3 (0.8945)  -0.0962 (0.1778) | 0.0646 (0.3208)  0.0327 (0.6123) | 0.0173 (0.8081)  -0.131 (0.0612) |
| Dust –current  –past | -0.0981 (0.0904)  -5.16E-3 (0.9170) | -0.121 (0.1125)  -0.0241 (0.7104) | -0.0421 (0.5401)  5.62E-3 (0.9233) | -0.0727 (0.3326)  0.0514 (0.4198) |
| Other nano – current  –past | -0.0998 (0.1338)  -0.141 (0.0435) | -0.162 (0.0624)  -0.155 (0.0923) | 0.0308 (0.696)  -0.125 (0.1315) | 0.0123 (0.8869)  -0.0762 (0.4017) |

Abbreviations – CHM: cardiovascular health metric; CNT/F: carbon nanotubes or nanofibers; EC: elemental carbon; F: female PM: particulate matter; NSAID: non-steroidal anti-inflammatory drug; TEM: transmission electron microscopy; U/FP: ultrafine and fine particulate; UP: ultrafine particulate; W: white

**Table S10. Results of univariable linear regression modeling of other transformed CBC metrics (highlight indicates selected in “best model” by Schwartz Bayesian Criterion and considered as potential confounder in multiple linear regression model with main exposure variables**)

|  | **β estimate (p)** | | |
| --- | --- | --- | --- |
| **Transformed metric🡪** | **ln-Platelets** | **Hemoglobin^2^** | **Hematocrit^2^** |
| EC–inhalable  ln(EC–inhalable) | 6.92E-5 (0.8867)  -2.86E-3 (0.6708) | -7.72E-3 (0.9259)  0.302 (0.7932) | -0.185 (0.7656)  -0.585 (0.9458) |
| EC-respirable  ln(EC-respirable) | 2.07E-3 (0.6081)  0.0139 (0.1823) | -0.204 (0.7687)  -1.84 (0.3038) | -2.81 (0.5870)  -13.1 (0.3262) |
| TEM-total mean  ln(TEM-total mean) | 3.87E-3 (0.9277)  -0.0104 (0.1848) | -4.93 (0.4990)  1.52 (0.2573) | -51.9 (0.3400)  11.3 (0.2619) |
| Sputum presence | -0.0811 (0.1248) | 9.76 (0.3052) | 86.3 (0.2243) |
| CNT/F duration emp | -5.43E-4 (0.9160) | 0.882 (0.3153) | 5.78 (0.3790) |
| ***Confounder variable – Personal characteristics*** | | | |
| Age | -1.77E-3 (0.2630) | -0.144 (0.5099) | -0.390 (0.8153) |
| Sex (F:M) | 0.141 (0.0058) | -54.8 (<0.0001) | -392 (<0.0001) |
| Race (W,non-Hisp:Oth) | 0.128 (0.0334) | 17.0 (0.0404) | 131 (0.0397) |
| Cigarette pack-years | 1.51E-3 (0.3459) | -0.199 (0.4676) | -1.28 (0.5315) |
| CHM score (high:low) | -0.0655 (0.1152) | 18.0 (0.0104) | 115 (0.0296) |
| Alcohol-drinks per week | -8.79E-3 (0.0295) | 0.0811 (0.9077) | 1.44 (0.7822) |
| Current resp infection | 0.0800 (0.2924) | -32.2 (0.0122) | -277 (0.0037) |
| Current NSAID use | -0.0152 (0.748) | 0.177 (0.9826) | 15.0 (0.8034) |
| Anticholesterol med use | 0.086 (0.1596) | -15.3 (0.1439) | -94.5 (0.2278) |
| Antihypertensive use | 0.0523 (0.3657) | -13.4 (0.1745) | -106 (0.1493) |
| ***Confounder variable – Workplace exposures*** | | | |
| Mean U/FP counts | 2.35E-6 (0.0518) | 1.06E-7 (0.9996) | 4.15E-4 (0.7900) |
| Mean UP counts | 2.42E-6 (0.1061) | 7.43E-5 (0.7729) | 8.89E-4 (0.6443) |
| Mean PM-2.5 mass | 1.95E-3 (0.4119) | -0.343 (0.3992) | -1.49 (0.6256) |
| Solvent – current  –past | -0.0554 (0.1833)  -0.0646 (0.1194) | 8.74 (0.2196)  1.90 (0.7903) | 40.4 (0.4492)  -26.6 (0.6177) |
| Polymers – current  –past | -0.0454 (0.3300)  -0.0483 (0.2943) | 13.5 (0.0893)  -0.814 (0.9180) | 64.0 (0.2826)  -30.5 (0.6056) |
| Dust –current  –past | -0.0522 (0.2879)  -0.0122 (0.7702) | 4.79 (0.5690)  8.77 (0.2179) | 42.6 (0.4982)  75.2 (0.1569) |
| Other nano – current  –past | 0.0292 (0.6049)  -0.0195 (0.7436) | 3.19 (0.7412)  17.4 (0.0853) | -18.0 (0.8034)  104 (0.1697) |

Abbreviations – CHM: cardiovascular health metric; CNT/F: carbon nanotubes or nanofibers; EC: elemental carbon; F: female PM: particulate matter; NSAID: non-steroidal anti-inflammatory drug; TEM: transmission electron microscopy; U/FP: ultrafine and fine particulate; UP: ultrafine particulate; W: white
