# Supplementary material for: Association of pulmonary, cardiovascular, and hematologic metrics with carbon nanotube and nanofiber exposure among U.S. workers: a cross-sectional study
Source: Part Fibre Toxicol. 2018 May 16;15:22. doi: 10.1186/s12989-018-0258-0 (PMC5956815; doi:10.1186/s12989-018-0258-0)
Supplement: Supplementary file 2 — Questionnaire. (DOCX 37 kb) [file 12989_2018_258_MOESM2_ESM.docx]

**Additional File 2: Questionnaire**

Association of pulmonary, cardiovascular, and hematologic metrics with carbon nanotube and nanofiber exposure among U.S. workers: A cross-sectional study

Mary K. Schubauer-Berigan, Matthew M. Dahm, Aaron Erdely, John D. Beard, M. Eileen Birch, Douglas E. Evans, Joseph E. Fernback, Robert R. Mercer, Stephen J. Bertke, Tracy Eye, Marie A. de Perio

***Questionnaire administered to each participant***

**INTERVIEWER: PLEASE READ THIS TO PARTICIPANT PRIOR TO BEGINNING INTERVIEW**

**If you are concerned about providing sensitive information or have questions about participating, feel free to discuss this with me now. If you choose not to participate, you can quit now or at any time during the study. You may decline to answer any question, for any reason, and continue with the rest of the interview.**

**The purpose of this questionnaire is to obtain information about factors other than carbon nanotube or nanofiber exposures at your present job that might affect your health. The subjects that will be covered are your personal characteristics (that is, your birth date, race or ethnicity, and sex), your work history involving exposure to carbon nanotubes or nanofibers or other chemicals, dusts or fumes at** *(name of company under study)***, your hobbies, your history of certain illnesses, your use of certain prescription medications, your tobacco smoking habits, and your alcohol consumption patterns.**

**The information you provide will be protected under the Federal Privacy Act. Your personal information will not be shared with anyone in a way that allows you to be identified. We ask about your Social Security Number (SSN) so that we can link information about your health to your work history records at your company. However, you do not have to provide us your SSN.**

1. STUDY ID NUMBER: |__|__|__|__|__| (completed by NIOSH)

**2. NAME**: a. |__|__|__|__|__|__|__|__|__|__|__|__|__|__|__| b. |__| c. |__|__|__|__|__|__|__|__|__|__|__|__|__|__|__|__|__|

(first) (middle) (last)

**3. ADDRESS**: a. |__|__|__|__|__|__|__|__|__|__|__|__|__|__|__|__|__|__|__|__|__|

(street)

b. |__|__|__|__|__|__|__|__|__|__|__|__|__|__|__|__|__|__|__|__|__| c. |__|__| d. |__|__|__|__|__|

(city) (state) (zip code)

**4. TELEPHONE**: |__|__|__| / |__|__|__| - |__|__|__|__|

(area code)

**5. SOCIAL SECURITY NUMBER**: |__|__|__| - |__|__| - |__|__|__|__|

PERSONAL AND MEDICAL HISTORY

6. Interviewer’s Initials |__|__|__|

7. Interview date: a. Day |__|__| b. Month |__|__| c. Year |__|__|__|__|

**FIRST, I WOULD LIKE TO ASK YOU SOME QUESTIONS ABOUT YOUR PERSONAL CHARACTERISTICS.**

8. **WHAT IS YOUR DATE OF BIRTH?** a. Day |__|__| b. Month |__|__| c. Year |__|__|__|__|

9. **WHAT IS YOUR SEX OR GENDER?**

|__| 0. Male; 1. Female; 8. Refused; 9. Other)

10. **WOULD YOU DESCRIBE YOUR RACE AS:** *(Read choices & indicate response to each)*

a. |__| White (0. No; 1. Yes; 8. Refused; 9. Don’t know)

b. |__| Black or African American (0. No; 1. Yes; 8. Refused; 9. Don’t know)

c. |__| American Indian or Alaska Native (0. No; 1. Yes; 8. Refused; 9. Don’t know)

d. |__| Asian (0. No; 1. Yes; 8. Refused; 9. Don’t know)

e. |__| Native Hawaiian or other Pacific Islander (0. No; 1. Yes; 8. Refused; 9. Don’t know)

11. **WOULD YOU DESCRIBE YOURSELF AS OF HISPANIC OR LATINO/LATINA ORIGIN?**

|__| (0. No; 1. Yes; 8. Refused; 9. Don’t know)

12. Current height? a. |__| feet b. |__|__| inches

13. Current weight? |__|__|__| pounds (888=refused to answer, 999=don’t know)

14. What is your level of schooling? |__| (*0 = None 1 = 1-7 years, 2 = elementary school graduate, 3=9-11 years, 4= high school graduate, 5= vocational school, 6=some college, 7=college graduate, 8=postgraduate, 9=refused)*

**NOW I WOULD LIKE TO ASK YOU ABOUT SYMPTOMS THAT PERTAIN MOSTLY TO YOUR CHEST. PLEASE ANSWER YES OR NO IF POSSIBLE. IF A QUESTION DOES NOT APPEAR TO BE APPLICABLE TO YOU, LET ME KNOW. (For questions 15-48, if the participant is in doubt about whether his or her answer is yes or no, record no.)**

**15. COUGH**

a. Do you usually have a cough? (Count a cough with first smoke or on first going out-of-doors. |__| 0. No; 1. Yes; 8. Ref

Exclude clearing of throat.) [If no, skip to question 15c.]

b. Do you usually cough as much as 4 to 6 times a day, 4 or more days out of the week? |__| 0. No; 1. Yes; 8. Ref

c. Do you usually cough at all on getting up, or first thing in the morning? |__| 0. No; 1. Yes; 8. Ref

d. Do you usually cough at all during the rest of the day or at night? |__| 0. No; 1. Yes; 8. Ref

IF YES TO ANY OF THE ABOVE (15a-d), ANSWER THE FOLLOWING:

IF NO TO ALL, SKIP TO 16a.

e. Do you usually cough like this on most days for 3 consecutive months or more during the year? |__| 0. No; 1. Yes; 8. Ref

f. For how many years have you had this cough? |__|__| Number of years

================================================================================

**16. PHLEGM**

a. Do you usually bring up phlegm from your chest? |__| 0. No; 1. Yes; 8. Ref

(Count phlegm with the first smoke or on first going out-of-doors. Exclude phlegm from the nose. Count swallowed phlegm)

[If no, skip to 16c.]

b. Do you usually bring up phlegm like this as much as twice a day, 4 or more days out of the week? |__| 0. No; 1. Yes; 8. Ref

c. Do you usually bring up phlegm at all on getting up or first thing in the morning? |__| 0. No; 1. Yes; 8. Ref

d. Do you usually bring up phlegm at all during the rest of the day or at night? |__| 0. No; 1. Yes; 8. Ref

IF YES TO ANY OF THE ABOVE (16a-d), ANSWER THE FOLLOWING:

IF NO TO ALL, SKIP TO 17a.

e. Do you bring up phlegm like this on most days for 3 consecutive months or more during the year? |__| 0. No; 1. Yes; 8. Ref

f. For how many years have you had trouble with phlegm? |__|__| Number of years

==============================================================================

**17 EPISODES OF COUGH AND PHLEGM**

a. Have you had periods or episodes of (increased*) cough and phlegm lasting for 3 weeks or more |__| 0. No; 1. Yes; 8. Ref

each year?

*(For individuals who usually have cough and/or phlegm)

IF YES TO 17a:

b. For how long have you had at least 1 such episode per year? |__|__| Number of years

==============================================================================

**WHEEZING**

18a. Does your chest ever sound wheezy or whistling:

1. When you have a cold? |__| 0. No; 1. Yes; 8. Ref

2. Occasionally apart from colds? |__| 0. No; 1. Yes; 8. Ref

3. Most days or nights? |__| 0. No; 1. Yes; 8. Ref

IF YES TO 1, 2, OR 3 IN 18a:

b. For how many years has this been present? |__|__| Number of years

19a. Have you ever had an ATTACK of wheezing that has made you feel short of breath? |__| 0. No; 1. Yes; 8. Ref

IF YES TO 19a, ANSWER 19b-d (IF NO, SKIP TO 20):

b. How old were you when you had your first such attack? |__|__|__| Age in years (888 Ref; 999 Don’t know)

c. Have you had 2 or more such episodes? |__| 0. No; 1. Yes; 8. Ref

d. Have you ever required medicine or treatment for the(se) attack(s)? |__| 0. No; 1. Yes; 8. Ref

==============================================================================

**BREATHLESSNESS**

20a. Are you disabled from walking? |__| 0. No; 1. Yes; 8. Ref

IF YES TO 20a, answer 20b (IF NO OR REFUSED, SKIP TO 21a)

b. Is this disability due to heart or lung disease? |__| 0. No; 1. Yes; 8. Ref

IF NO to 20b, SKIP TO 22

21a. Do you have shortness of breath when hurrying on the level or walking up a slight hill? |__| 0. No; 1. Yes; 8. Ref

IF YES TO 21a, ANSWER 21b-e (IF NO, SKIP TO 22):

b. Do you have to walk slower on the level than people of your age because of breathlessness? |__| 0. No; 1. Yes; 8. Ref

c. Do you ever have to stop for breath when walking at your own pace on the level? |__| 0. No; 1. Yes; 8. Ref

d. Do you ever have to stop for breath after walking about 100 yards (or after a few minutes) on |__| 0. No; 1. Yes; 8. Ref

the level?

e. Are you too breathless to leave the house or breathless on dressing or undressing? |__| 0. No; 1. Yes; 8. Ref

==============================================================================

**CHEST COLDS AND CHEST ILLNESSES**

22. If you get a cold, does it usually go to your chest? (Usually means more than 1/2 the time) |__| 0. No; 1. Yes; 7. No colds; 8. Ref

23a. During the past 3 years, have you had any chest illnesses that have kept you off work, indoors |__| 0. No; 1. Yes; 8. Ref

at home, or in bed?

IF YES TO 23a, ANSWER 23b-c (IF NO, SKIP TO 23d):

b. Did you produce phlegm with any of these chest illnesses? |__| 0. No; 1. Yes; 8. Ref

c. In the last 3 years, how many such illnesses, with (increased) phlegm, did you have which _____Number of illnesses

lasted a week or more?

d. Do you currently have a cold or other upper airway infectious disease? |__| 0. No; 1. Yes; 8. Ref

**==============================================================================**

**PAST ILLNESSES**

24a. Have you ever had pneumonia (include bronchopneumonia)? |__| 0. No; 1. Yes; 8. Ref

IF YES TO 24a, ANSWER b and c (IF NO, SKIP TO 25a):

b. Was it confirmed by a doctor? |__| 0. No; 1. Yes; 8. Ref

c. At what age did you first have it? |__|__|__| Age in years (888 Ref; 999 Don’t know)

25a. Have you ever had hay fever or respiratory allergies? |__| 0. No; 1. Yes; 8. Ref

IF YES TO 25a, ANSWER b and c (IF NO, SKIP TO 26a):

b. Was it confirmed by a doctor? |__| 0. No; 1. Yes; 8. Ref

c. At what age did it start? |__|__|__| Age in years (888. Ref; 999 Don’t know)

26a. Have you ever had chronic bronchitis? |__| 0. No; 1. Yes; 8. Ref

IF YES TO 26a, ANSWER b-d (IF NO, SKIP TO 27a):

b. Do you still have it? |__| 0. No; 1. Yes; 8. Ref

c. Was it confirmed by a doctor? |__| 0. No; 1. Yes; 8. Ref

d. At what age did it start? |__|__|__| Age in years (888 Ref; 999 Don’t know)

27a. Have you ever had emphysema? |__| 0. No; 1. Yes; 8. Ref

IF YES TO 27a, ANSWER b-d (IF NO, SKIP TO 28a):

b. Do you still have it? |__| 0. No; 1. Yes; 8. Ref

c. Was it confirmed by a doctor? |__| 0. No; 1. Yes; 8. Ref

d. At what age did it start? |__|__|__| Age in years (888 Ref; 999 Don’t know)

28a. Have you ever had asthma? |__| 0. No; 1. Yes; 8. Ref

IF YES TO 28a, ANSWER 28b-e (IF NO, SKIP TO 29a):

b. Do you still have it? |__| 0. No; 1. Yes; 8. Ref

c. Was it confirmed by a doctor? |__| 0. No; 1. Yes; 8. Ref

d. At what age did it start? |__|__|__| Age in years (888 Ref; 999 Don’t know)

e. If you no longer have it, at what age did it stop? |__|__|__| Age in years (888 Ref; 999 Don’t know)

29. Have you ever had:

a. Any other chest illnesses? |__| 0. No; 1. Yes; 8. Ref

If yes, please specify ____________________________________________

b. Any chest operations? |__| 0. No; 1. Yes; 8. Ref

If yes, please specify ____________________________________________

c. Any chest injuries? |__| 0. No; 1. Yes; 8. Ref

If yes, please specify ____________________________________________

30a. Has a doctor ever told you that you had heart trouble? |__| 0. No; 1. Yes; 8. Ref

IF YES to 30a, ANSWER 30b-c (IF NO, GO TO 31a):

b. Have you had treatment for heart trouble in the past 10 years? |__| 0. No; 1. Yes; 8. Ref

c. Has a doctor ever told you that you have had a heart attack? |__| 0. No; 1. Yes; 8. Ref

31a. Has a doctor ever told you that you have high blood pressure? |__| 0. No; 1. Yes; 8. Ref

IF YES to 31a, ANSWER 31b (IF NO, GO TO 32a):

b. Have you had any treatment for high blood pressure (hypertension) in the past 10 years? |__| 0. No; 1. Yes; 8. Ref

==============================================================================

**NOW I WOULD LIKE TO ASK YOU ABOUT CERTAIN OTHER ILLNESSES OR HEALTH CONDITIONS YOU MAY HAVE HAD (Note: affirmative responses to any questions 40-45, or stroke or heart attack within the past 3 months (questions 30c, 33a and 46), involve exclusions for spirometry)**:

| HAVE YOU EVER HAD:  (Did you have any other?) | WHEN WAS THIS CONDITION FIRST DIAGNOSED? |
| --- | --- |
| 32a. **Malignant tumor or cancer?** (including leukemia or lymphoma)  \|__\| 0. No; 1. Yes; 8. Ref  *(Describe)*: | b. Mo \|__\|__\| c. Yr \|__\|__\|__\|__\| |
| 33a. **Diagnosed heart disease?** *(Describe)*:  \|__\| 0. No; 1. Yes; 8. Ref | b. Mo \|__\|__\| c. Yr \|__\|__\|__\|__\| |
| 34a. **Diabetes?**  \|__\| 0. No; 1. Yes; 8. Ref | b. Mo \|__\|__\| c. Yr \|__\|__\|__\|__\| |
| 35a. **Kidney disease?**  *(Describe):*  \|__\| 0. No; 1. Yes; 8. Ref | b. Mo \|__\|__\| c. Yr \|__\|__\|__\|__\| |
| 36a. **Cystic fibrosis?**  \|__\| 0. No; 1. Yes; 8. Ref | b. Mo \|__\|__\| c. Yr \|__\|__\|__\|__\| |
| 37a. **Scleroderma?**  \|__\| 0. No; 1. Yes; 8. Ref | b. Mo \|__\|__\| c. Yr \|__\|__\|__\|__\| |
| 38a. **Lupus (systemic lupus erythromatosus)?**  \|__\| 0. No; 1. Yes; 8. Ref | b. Mo \|__\|__\| c. Yr \|__\|__\|__\|__\| |
| 39a. **Any other autoimmune disease?** *(Describe):*  \|__\| 0. No; 1. Yes; 8. Ref | b. Mo \|__\|__\| c. Yr \|__\|__\|__\|__\| |
| 40a. **Eye surgery?** (other than cosmetic surgery on the eyelid or skin around the eye)  \|__\| 0. No; 1. Yes; 8. Ref | b. Mo \|__\|__\| c. Yr \|__\|__\|__\|__\| |
| 41a. **Open chest or abdominal surgery?**  \|__\| 0. No; 1. Yes; 8. Ref | b. Mo \|__\|__\| c. Yr \|__\|__\|__\|__\| |
| 42. **Did you or anyone in your household have tuberculosis in the past year?**  \|__\| 0. No; 1. Yes; 8. Ref |  |
| 43. **Has a doctor or other health professional told you that you had an aneurysm?**  \|__\| 0. No; 1. Yes; 8. Ref |  |
| 44. **Has a doctor or other health professional told you that you had a collapsed lung?**  \|__\| 0. No; 1. Yes; 8. Ref |  |
| 45. **Has a doctor or other health professional told you that you had a detached retina?**  \|__\| 0. No; 1. Yes; 8. Ref |  |
| 46a. **Has a doctor or other health professional told you that you had a stroke?**  \|__\| 0. No; 1. Yes; 8. Ref | b. Mo \|__\|__\| c. Yr \|__\|__\|__\|__\| |
| 47. **In the past month, have you coughed up blood?**  \|__\| 0. No; 1. Yes; 8. Ref |  |
| 48. **Are you currently taking any prescription or nonprescription medication, including aspirin?**  \|__\| 0. No; 1. Yes; 8. Ref  49. **Please list all prescription and non-prescription medication you are currently taking.** |  |
| **50. Are you currently pregnant?**  \|__\| 0. No; 1. Yes; 8. Ref 9. Don’t Know |  |

**NOW I WOULD LIKE TO ASK YOU ABOUT YOUR WORK HISTORY.**

51. **WHAT IS YOUR REGULAR WORK SHIFT?** *(If response is 4 or 8, skip to question 53)*

|__| 1. Days; 2. Evenings; 3. Nights; 4. Rotating; 8. Refused

52. **WHAT ARE YOUR REGULAR SHIFT HOURS?**

From a. |__|__|__|__|__|__| (incl. am/pm) to b. |__|__|__|__|__|__| (incl. am/pm)

53. **HOW MANY HOURS PER WEEK DO YOU USUALLY WORK?** |__|__| Hours (888 – Refused; 999 –Don’t Know)

**NOW I WOULD LIKE TO ASK YOU ABOUT THE JOBS YOU’VE HAD.**

**54. PLEASE DESCRIBE YOUR CURRENT JOB**.

| **a. WHAT IS THE NAME & LOCATION** *(City/State)* **OF** **THE** **COMPANY?** | **b. WHAT IS YOUR DEPART-MENT?** | **c. WHAT IS YOUR JOB TITLE?** | **d. WHEN DID YOU START WORKING IN THIS JOB?**  *(Month/Year)* | **e. WHAT ARE YOUR ACTIVITIES & DUTIES?** | **f. DESCRIBE ANY CNT OR CNF, CHEMICALS, DUSTS, OR FUMES, INCLUDING DIESEL EXHAUST THAT YOU HAVE BEEN EXPOSED TO IN THIS JOB.** |
| --- | --- | --- | --- | --- | --- |
|  |  |  | **Start:** \|__\|__\|/\|__\|__\|__\|__\| |  |  |

**55a. OTHER THAN YOUR CURRENT JOB, WHICH YOU JUST DESCRIBED, HAVE YOU WORKED IN ANY JOBS WHERE YOU WERE EXPOSED TO CARBON NANOTUBES (CNT) OR CARBON NANOFIBERS (CNF) OR CHEMICALS, DUSTS OR FUMES, INCLUDING DIESEL EXHAUST?**

0 |__| No *(Go to #56)*

1 |__| Yes *(complete table below)*

8 |__| Refused *(Go to #56)*

9 |__| Don’t know *(Go to #56)*

**PLEASE DESCRIBE THE OTHER JOB(S) YOU HAD WHERE YOU WERE EXPOSED TO CNT, CNF, OR CHEMICALS, DUSTS OR FUMES.**

| **b. WHAT WAS THE NAME & LOCATION** *(City/State)* **OF** **THE** **COMPANY?** | **c. WHAT WAS YOUR DEPART-MENT?** | **d. WHAT WAS YOUR JOB TITLE?** | **WHEN DID YOU e. START AND f. STOP WORKING IN THIS JOB?**  *(Month/Year)* | **g. WHAT WERE YOUR ACTIVITIES AND DUTIES?** | **h. DESCRIBE ANY CNT OR CNF, CHEMICALS, DUSTS, OR FUMES, INCLUDING DIESEL EXHAUST, THAT YOU WERE EXPOSED TO.** |
| --- | --- | --- | --- | --- | --- |
| i. |  |  | **Start:** \|__\|__\|/\|__\|__\|__\|__\|  **Stop:** \|__\|__\|/\|__\|__\|__\|__\| |  |  |
| ii. |  |  | **Start:** \|__\|__\|/\|__\|__\|__\|__\|  **Stop:** \|__\|__\|/\|__\|__\|__\|__\| |  |  |
| iii. |  |  | **Start:** \|__\|__\|/\|__\|__\|__\|__\|  **Stop:** \|__\|__\|/\|__\|__\|__\|__\| |  |  |
| iv. |  |  | **Start:** \|__\|__\|/\|__\|__\|__\|__\|  **Stop:** \|__\|__\|/\|__\|__\|__\|__\| |  |  |

56a. **NOW I WOULD LIKE TO ASK YOU ABOUT YOUR HOBBIES.**

**IN THE PAST 6 MONTHS, HAVE YOU HAD ANY HOBBIES WHERE YOU WORKED WITH ANY CHEMICALS OR HAD EXPOSURE TO CHEMICAL VAPORS, DUSTS OR FUMES, INCLUDING DIESEL EXHAUST?** **(SOME EXAMPLES MIGHT BE CARPENTRY OR FURNITURE REFINISHING, OR MAKING STAINED GLASS AS A HOBBY. INCLUDE ACTIVITIES ONLY IF YOU HAVE DONE THEM AT LEAST ONE HOUR A WEEK).**

0 |__| No *(Go to #56)*

1 |__| Yes *(Complete table below)*

8 |__| Refused *(Go to #56)*

9 |__| Don’t know *(Go to #56)*

| **b. WHAT IS THE EXACT NATURE OF THE HOBBY?** | **c. HOW MANY HOURS PER WEEK DID YOU DO THIS**  **IN THE PAST 6 MONTHS?** | **d. WHAT CHEMICALS, DUSTS, OR FUMES (INCLUDING DIESEL EXHAUST) ARE YOU EXPOSED TO IN THIS HOBBY?** |
| --- | --- | --- |
| i. |  |  |
| ii. |  |  |

**IN THE NEXT PART OF THE QUESTIONNAIRE, I WOULD LIKE TO ASK YOU ABOUT YOUR TOBACCO HISTORY AND YOUR ALCOHOL CONSUMPTION PATTERNS.**

| **DID YOU EVER:** | **b. DO YOU CURRENTLY:** | **c. WHAT AMOUNT, ON AVERAGE, DO/DID YOU SMOKE/USE PER DAY?** (one pack = 20 cigarettes) | **d-e. DURING WHICH YEARS DID YOU:** |
| --- | --- | --- | --- |
| **57a. SMOKE CIGARETTES** \|__\| 0. No; 8. Ref  (at least 100 in your lifetime) \|__\| 1. Yes🡪 | \|__\| 0. No; 1. Yes; 8. Ref | \|__\|__\|__\| cigarettes/day  or \|__\| packs/day | \|__\|__\|__\|__\| to \|__\|__\|__\|__\| |
| **58. SMOKE CIGARS** \|__\| 0. No; 8. Ref  (at least once/day for 6 months) \|__\| 1. Yes🡪 | \|__\| 0. No; 1. Yes; 8. Ref | \|__\|__\| cigars/day | \|__\|__\|__\|__\| to \|__\|__\|__\|__\| |
| **59. SMOKE A PIPE** \|__\| 0. No; 8. Ref  (at least once/day for 6 months) \|__\| 1. Yes🡪 | \|__\| 0. No; 1. Yes; 8. Ref | \|__\|__\| pipesful/day | \|__\|__\|__\|__\| to \|__\|__\|__\|__\| |
| **60. Chew tobacco or use snuff?** \|__\| 0. No;  (at least once/day for 6 months) \|__\| 1. Yes🡪  8. Ref | \|__\| 0. No; 1. Yes; 8. Ref | \|__\|__\| ounces /day | \|__\|__\|__\|__\| to \|__\|__\|__\|__\| |
| **61a. LIVE with a regular smoker? (daily for 6 months or more)**  \|__\| 0. No; 8. Ref  \|__\| 1. Yes🡪 | \|__\| 0. No; 1. Yes; 8. Ref |  | \|__\|__\|__\|__\| to \|__\|__\|__\|__\| |
|  |  |  |  |

62. Did you ever drink alcoholic beverages at least 12 or more times in a single year?

0 |__| No *(End interview)*  1 |__| Yes

8 |__| Refused *(End interview)*

63. How many years total did you drink at least 12 or more alcoholic beverages in a single year?

|__|__|__| Years, or 888 Refused, or 999 Don’t Know or year |__|__|__|__|to|__|__|__|__| or Age |__|__|to|__|__|

64. During this period, about how many drinks (cans or glasses of beer, glasses of wine, shots of hard liquor straight or in a mixed drink) did you usually have per week? |__|__|__| (number of drinks, or 888 Refused or 999 Don’t know)

| 65a. **OVER THE PAST 6 MONTHS, HAVE YOU CONSUMED, ON AVERAGE, AT LEAST 1 ALCOHOLIC BEVERAGE PER WEEK SUCH AS BEER, WINE, MIXED DRINKS, OR HARD LIQUOR?**  0 \|__\| No *(End interview)*  1 \|__\| Yes *(Continue)* --------------------->  2 \|__\| Chooses not to respond *(End interview)* | b. **PLEASE ESTIMATE HOW MANY TIMES, PER WEEK, YOU DRANK ALCOHOLIC BEVERAGES ON AVERAGE, OVER THE PAST 6 MONTHS,.**  \|__\|__\| per week |
| --- | --- |
|  | c. **PLEASE ESTIMATE THE NUMBER OF DRINKS YOU HAVE, ON AVERAGE, ON EACH OCCASION.**  \|__\|__\|__\| (drinks; 888 Refused; 999 Don’t know) |

Thank you! This completes the interview
