# Supplementary material for: Association of pulmonary, cardiovascular, and hematologic metrics with carbon nanotube and nanofiber exposure among U.S. workers: a cross-sectional study
Source: Part Fibre Toxicol. 2018 May 16;15:22. doi: 10.1186/s12989-018-0258-0 (PMC5956815; doi:10.1186/s12989-018-0258-0)
Supplement: Supplementary file 3 — Supplementary information. (DOCX 24 kb) [file 12989_2018_258_MOESM3_ESM.docx]

**Additional File 3: Supplementary information**

Association of pulmonary, cardiovascular, and hematologic metrics with carbon nanotube and nanofiber exposure among U.S. workers: A cross-sectional study

Mary K. Schubauer-Berigan, Matthew M. Dahm, Aaron Erdely, John D. Beard, M. Eileen Birch, Douglas E. Evans, Joseph E. Fernback, Robert R. Mercer, Stephen J. Bertke, Tracy Eye, Marie A. de Perio

**Table of Contents**

Description of spirometry procedures……………………………………………………………..... 2

Description of sputum induction and processing procedures….…………………………………. 3

Supplemental references…………………………………………………………………………….. 3

***Description of spirometry procedures***

Two participants were excluded using the following criteria: eye, chest, or abdominal surgery, or heart attack or stroke (within the past 3 months), tuberculosis exposure of self or household member, history of aneurysm, collapsed lung, or detached retina (NHANES 2008) and one eligible participant refused; 103 participants completed spirometry. A volume-based spirometer (OMI/Sensormedics 1022™, OMI Inc, Houston TX) and a standing position were used for all participants. A certified spirometry technician employed diligent coaching to ensure maximum inspiration and forced expiration during each test. We used standard acceptability criteria for start and end of spirometry tests (Miller et al. 2005): no cough in the first second or excessive hesitation. In addition, a rise time to peak flow of <120 msec and volume at peak flow at <35% of FVC were required. Standard end-of-test criteria were used (Miller et al. 2005). Tracings that did not meet acceptability criteria but provided useable information were used in relevant analyses. At least three (and up to eight) spirometry maneuvers were conducted per subject, until three tests met criteria for acceptable start and end of test and acceptable repeatability of the FVC and FEV1 (Miller et al. 2005). Spirometer volume calibration was checked daily, and a linear volume calibration check conducted quarterly.

We used OMI™ spirometry software to calculate relevant parameters, including back-extrapolated volume, and percent predicted (PP) values for FVC, FEV1/FVC% (using the largest valid FEV1 and FVC), FEF25-75%, and PEF. All values were corrected for pressure differences at body compared to ambient temperature. Values were compared to the “lower limit of normal” (LLN), based on age-, height-, sex- and (for white, African-American, and Hispanic subjects) race/ethnicity-specific NHANES population data from Hankinson et al. (1999). For Asian subjects, we used an adjustment factor of 0.94 based on white subjects (Miller et al. 2005; Hankinson et al. 2010). Spirometry metrics were interpreted clinically using recommendations of the American Thoracic Society (Pellegrino et al. 2005); in statistical analyses, we used the PP value (based on age, height, sex, and race/ethnicity) as the outcome metric for FVC, FEV1/FVC, FEF25-75%, and PEF.

***Description of sputum induction and processing procedures***

Contraindications for sputum induction included: any contraindication for spirometry, pregnancy, use of beta-blockers, cardiac arrhythmia or angina, surgery or pneumothorax within the past three months, or baseline FEV1<60% of predicted. Eligible study participants inhaled, for 12 minutes in total, a sterile isotonic saline solution aerosolized by a DeVilbiss™ compressed-air generated nebulizer. Study participants sanitized hands, donned gloves, expelled nasal mucus, and rinsed their oral cavity before beginning. They then breathed aerosolized saline through the mouthpiece and, every two minutes, were asked to remove the mouthpiece, spit saliva into a cup, take a deep breath through the mouthpiece and then expectorate into a sputum cup. After six minutes of breathing through the nebulizer, the spirometry technician measured the FEV1 of the participant to ensure that it did not drop by 20% or more compared to baseline (none did). Breathing through the nebulizer continued for three more two-minute cycles, each followed by spitting into the saliva cup and expectoration into the sputum cup. We sought to collect at least 2 mL of sputum from each participant. The entire sputum specimen from each participant was diluted with an equal volume of 10% Sputolysin® (Sigma-Aldrich Co. LLC., St. Louis, MO) and mixed several times by pipette aspiration. The mucolytic suspension was incubated for 15 minutes in a shaking water bath (160 rotations per minute) at 37ºC, mixing the sample every 5 minutes. After incubation, the solution was centrifuged at 500xg for 10 minutes at 4ºC. The supernatant (acellular fraction) was pipetted into a clean tube, mixed well, aliquoted into cryovials, and held at -20ºC. The pellet containing the cellular fraction was resuspended in a cytology fixative (Saccomanno Fluid™, Sigma-Aldrich Co. LLC., St. Louis, MO), at a >3:1 ratio and placed on ice. At the end of each day, all sputum specimens were shipped to the NIOSH facility in Morgantown, WV. Acellular fractions were stored at -80◦C Cellular fractions were stored at 4ºC.

Supplemental References

Hankinson JL, Odencrantz JR, Fedan KB. 1999. Spirometric reference values from a sample of the general US population. Am J Respir Crit Care Med 159:179-187.

Hankinson JL, Kawut SM, Shahar E, Smith LJ, Stukovsky KH, Barr RG. 2010. Performance of American Thoracic Society-recommended spirometry reference values in a multiethnic sample of adults: the multi-ethnic study of atherosclerosis (MESA) lung study. Chest 137:138-45.

Miller MR, Crapo R, Hankinson J, Brusasco V, Burgos F, Casaburi R, Coates A, Enright P, van der Grinten CPM, Gustafsson P, Jensen R, Johnson DC, MacIntyre N, McKay R, Navajas D, Pedersen OF, Pellegrino R, Viegi G, Wanger J. 2005. General considerations for lung function testing. Eur Respir J 26:153-161.

National Health and Nutrition Examination Survey (NHANES). Respiratory Health Spirometry Procedures Manual. http://www.cdc.gov/nchs/data/nhanes/nhanes_07_08/spirometry.pdf (accessed February 7, 2011). January 2008, 76 pp.

Pellegrino R, Viegi G, Brusasco V, Crapo RO, Burgos F, Casaburi R, Coates A, van der Grinten CPM, Gustafsson P, Hankinson J, Jensen R, Johnson DC, MacIntyre N, McKay R, Miller MR, Navajas D, Pedersen OF, Wanger J. 2005. Interpretive strategies for lung function tests. Eur Respir J 26:948-968.
